# Interspecies exciton interactions lead to enhanced nonlinearity of dipolar excitons and polaritons in MoS_2_ homobilayers

**DOI:** 10.1038/s41467-023-39358-9

**Published:** 2023-06-27

**Authors:** Charalambos Louca, Armando Genco, Salvatore Chiavazzo, Thomas P. Lyons, Sam Randerson, Chiara Trovatello, Peter Claronino, Rahul Jayaprakash, Xuerong Hu, James Howarth, Kenji Watanabe, Takashi Taniguchi, Stefano Dal Conte, Roman Gorbachev, David G. Lidzey, Giulio Cerullo, Oleksandr Kyriienko, Alexander I. Tartakovskii

**Affiliations:** 1 Department of Physics and Astronomy, The University of Sheffield, Sheffield, S3 7RH UK; 2 Dipartimento di Fisica, Politecnico di Milano, Piazza Leonardo da Vinci, 32, Milano, 20133 Italy; 3 Department of Physics, University of Exeter, Stocker Road, Exeter, EX4 4PY UK; 4 RIKEN Center for Emergent Matter Science, Wako, Saitama 351-0198 Japan; 5 Department of Mechanical Engineering, Columbia University, NY 10027 New York, USA; 6 Department of Physics and Mathematics, University of Hull, Rober Blackburn, Hull HU6 7RX UK; 7 National Graphene Institute, University of Manchester, Manchester, UK; 8 Department of Physics and Astronomy, University of Manchester, Manchester, UK; 9 Research Center for Electronic and Optical Materials, National Institute for Materials Science, 1-1 Namiki, Tsukuba, 305-0044 Japan; 10 Research Center for Materials Nanoarchitectonics, National Institute for Materials Science, 1-1 Namiki, Tsukuba, 305-0044 Japan

**Keywords:** Two-dimensional materials, Microresonators, Two-dimensional materials

## Abstract

Nonlinear interactions between excitons strongly coupled to light are key for accessing quantum many-body phenomena in polariton systems. Atomically-thin two-dimensional semiconductors provide an attractive platform for strong light-matter coupling owing to many controllable excitonic degrees of freedom. Among these, the recently emerged exciton hybridization opens access to unexplored excitonic species, with a promise of enhanced interactions. Here, we employ hybridized interlayer excitons (hIX) in bilayer MoS_2_ to achieve highly nonlinear excitonic and polaritonic effects. Such interlayer excitons possess an out-of-plane electric dipole as well as an unusually large oscillator strength allowing observation of dipolar polaritons (dipolaritons) in bilayers in optical microcavities. Compared to excitons and polaritons in MoS_2_ monolayers, both hIX and dipolaritons exhibit ≈ 8 times higher nonlinearity, which is further strongly enhanced when hIX and intralayer excitons, sharing the same valence band, are excited simultaneously. This provides access to an unusual nonlinear regime which we describe theoretically as a mixed effect of Pauli exclusion and exciton-exciton interactions enabled through charge tunnelling. The presented insight into many-body interactions provides new tools for accessing few-polariton quantum correlations.

## Abstract (web summary)

In semiconductors, accessing nonlinear interactions between excitons strongly coupled to light will be key for quantum technologies. Here, in atomic bilayers of MoS_2_, new types of excitons are discovered showing strong inter-excitonic interactions.

## Introduction

Excitons in two-dimensional transition metal dichalcogenides (TMDs) have large oscillator strengths and binding energies^1^, making them attractive as a platform for studies of strong light-matter coupling in optical microcavities^2–5^. A variety of polaritonic states have been realised using monolayers of MX_2_ (M=Mo, W; X=S, Se) embedded in tunable^3,5–7^ and monolithic microcavities^8–12^.

One of the central research themes in polaritonics is the study of nonlinear interactions leading to extremely rich phenomena such as Bose-Einstein condensation^13,14^, polariton lasing^15,16^ or optical parametric amplification^17^. Polaritons formed from tightly bound neutral intralayer excitons in TMDs are not expected to show strong nonlinearity. However, pronounced nonlinear behaviour was observed for trion polaritons^7,18^ and Rydberg polaritons^19^. Enhanced nonlinearity can be achieved by employing excitonic states with a physically separated electron and hole, e.g. in adjacent atomic layers^20^ or quantum wells^21–25^. Such interlayer excitons have a large out-of-plane electric dipole moment, and thus can strongly mutually interact^26^. Typically, however, interlayer or ‘spatially indirect’ excitons possess low oscillator strength^20,27^. Thus, in order to strongly couple to cavity photons, hybridization with high-oscillator-strength intralayer excitons is required^11,22,24,25,28^.

An attractive approach for realization of dipolar excitons and polaritons is to employ the recently discovered exciton hybridization in MoS_2_ bilayers^29,30^. This approach allows realization of uniform samples suitable for the observation of macroscopic many-body phenomena^31^. Interlayer excitons unique to bilayer MoS_2_ possess a large oscillator strength, comparable to that of the intralayer exciton, arising from interlayer hybridization of valence band states, aided by a favourable orbital overlap and a relatively small spin-orbit splitting among semiconducting TMDs^29^. Such hybridized interlayer excitons (hIX) are highly tunable using out-of-plane electric field^32,33^ and their valley degree of freedom persists up to room temperature^34^.

Here we use hIXs in bilayer MoS_2_ to realize highly nonlinear excitonic and dipolaritonic effects. We unravel a previously unexplored interaction regime involving intra- and interlayer excitons stemming from the fermionic nature of the charge carriers in a valence band shared between different excitonic species. This regime, accessible using broadband excitation resonant with both hIX and intralayer exciton transitions, provides strong (up to 10 times) enhancement of the exciton nonlinearity, already enhanced by up to 8 times in MoS_2_ bilayers compared with monolayers. We support our experimental findings with a theoretical discussion of nonlinear contributions to energy shifts and exciton broadening, which become enabled in the presence of charge tunnelling.

## Results

### Inter- and intra-layer exciton hybridization in bilayer MoS_2_

**Fig. 1:**
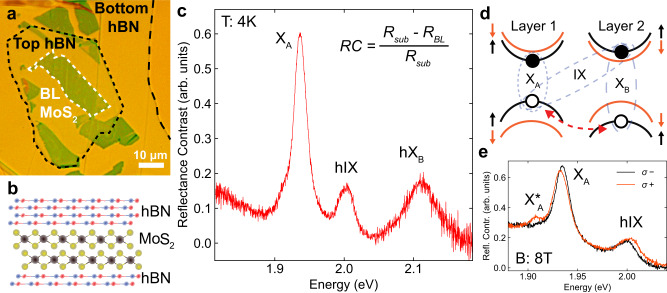
Homobilayer MoS_2_ and its optical response. **a** Bright field microscope image of an encapsulated BL MoS_2_ transferred on top of a DBR. **b** Schematic side-view of the fabricated heterostructure comprising a BL MoS_2_ sandwiched between few-layer hBN. **c** Reflectance contrast (RC) spectrum of the sample measured at low temperature (4 K) showing three distinct absorption features at 1.937 eV, 2.004 eV and 2.113 eV for X_A_, hIX and hX_B_, respectively. The measured linewidths for X_A_, hIX, and hX_B_ are 20, 23 and 64 meV, respectively. RC is calculated using the formula in the top-right corner of the graph. **d** Sketch of the conduction and valence bands in two adjacent layers of MoS_2_, displaying the allowed optical transitions of A and B direct intralayer excitons (X_A_ and X_B_) and interlayer excitons (IX) for spin-up states (black lines) at the K point in the bilayer momentum space. IX hybridizes with X_B_ through the hole tunnelling between the two layers (red dashed arrow). At the K' point of the bilayer Brillouin zone, the same configuration applies for the states with the opposite spins. **e** RC spectra of excitons in BL MoS_2_ detected in two circular polarizations in an out-of-plane magnetic field of 8 T at *T*=4 K. Zeeman shifts of opposite signs are observed for X_A_ and hIX. The absorption peak of the charged intralayer exciton (X${}_{{{{{{{{\rm{A}}}}}}}}}^{*}$) shows near unity circular polarization.

Our heterostructure samples consists of a MoS_2_ bilayer (BL) sandwiched between hBN and placed on a distributed Bragg reflector (DBR). Figure 1a shows a bright field microscope image of the encapsulated BL MoS_2_. A sketch of the side view of the device is displayed in Fig. 1b. The reflectance contrast (RC) spectrum of the studied MoS_2_ bilayer, displayed in Fig. 1c, shows three peaks: the intralayer neutral excitons X_A_ at at 1.937 eV (see Fig. 1d), hybridized interlayer exciton hIX at 2.004 eV and hybridized B-exciton at 2.113 eV. Due to the quantum tunnelling of holes, B-excitons hybridize with an interlayer exciton (IX) (Fig. 1d), which is a direct transition in the bilayer momentum space^29^. The ratio of the integrated intensities of X_A_ and hIX is 4.5. Based on these data, we estimate the electron-hole separation *d* = 0.55 nm (see details in Supplementary Note S1) in agreement with previous studies^34^. We further confirm the nature of the hIX states by placing the BL MoS_2_ in magnetic field where the valley degeneracy is lifted (Fig. 1e). In agreement with recent studies^33,35^, we measure a Zeeman splitting with an opposite sign and larger magnitude in hIX compared with X_A_ (−3.5 versus 1.5 meV at 8 Tesla).

### Hybridised interlayer excitons in the strong-coupling regime

**Fig. 2:**
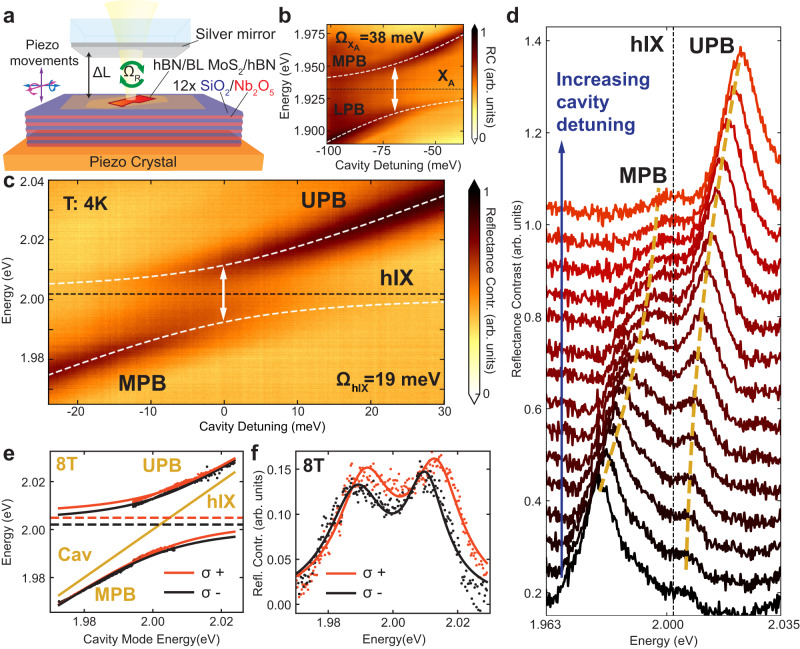
Strong exciton-photon coupling in MoS_2_ bilayers. **a** Schematics of the tunable open microcavity composed of a bottom DBR and a top semi-transparent silver mirror. **b**, **c** Low temperature (4K) RC spectra measured as a function of the cavity-exciton detuning (Δ = *E*_cav_ − *E*_exc_) for cavity scans across **b** X_*A*_ and **c** hIX energies. White dotted lines show the fitting obtained using the coupled oscillator model providing the Rabi splittings Ω_hIX_ = 19 meV and ${\Omega }_{{{{{{{{{\rm{X}}}}}}}}}_{{{{{{{{\rm{A}}}}}}}}}}=38$ meV. **d** RC spectra measured for the cavity-exciton detunings in the vicinity of the anticrossing between hIX and the cavity mode. **e** Dipolariton dispersion measured with circularly polarized detection for 8 T magnetic field. The orange and black solid curves are the coupled oscillator model fits for *σ*^+^ and *σ*^−^ detection, respectively. The positions of the Zeeman-split hIX peaks are shown by dashed lines. **f** *σ*^+^ (orange) and *σ*^−^ (black) RC spectra measured at 8 T at the hIX-cavity anticrossing. Fitting with two Lorentzians (solid lines) is shown.

We study the strong coupling regime in a tunable planar microcavity (Fig. 2a) formed by a silver mirror and a planar DBR^3^. RC scans as a function of the cavity mode detuning Δ = *E*_*cav*_ − *E*_*exc*_, where *E*_*cav*_ and *E*_*exc*_ are the cavity mode and the corresponding exciton energy, respectively, are shown in Fig. 2b, c. Characteristic anticrossings of the cavity mode with X_*A*_ and hIX are observed, resulting in lower, middle and upper polariton branches (LPB, MPB, and UPB, respectively). The extracted Rabi splittings are ${\Omega }_{{{{{{{{{\rm{X}}}}}}}}}_{{{{{{{{\rm{A}}}}}}}}}}=38$ meV for X_A_ and Ω_hIX_ = 19 meV for hIX (Supplementary Note S2). Figure 2d shows the RC spectra in the vicinity of the anticrossing with hIX, providing a more detailed view of the formation of the MPB and UPB. The intensity of the polariton peaks is relatively low for the states with a high exciton fraction at positive (negative) cavity detunings for the MPB (UPB). As the Rabi splitting scales as a square root of the oscillator strength, the ratio ${\Omega }_{{{{{{{{{\rm{X}}}}}}}}}_{{{{{{{{\rm{A}}}}}}}}}}/{\Omega }_{{{{{{{{\rm{hIX}}}}}}}}}=2$ is in a good agreement with the RC data for integrated intensities of X_*A*_ and hIX. From the Rabi splitting ratio we can estimate the tunnelling constant *J* leading to the B-exciton hybridization. The corresponding coefficient is *J* = 48 meV (see Supplementary Note S1 for details), matching the density functional theory predictions^29^. In polarization-resolved cavity scans in an out-of-plane magnetic field (Fig. 2e, f), similarly to hIX behaviour, we observe opposite and larger Zeeman splitting for dipolaritons relative to the intralayer polaritons (see Supplementary Fig. S4). Chiral dipolariton states are observed distinguished by their opposite circular polarization (Fig. 2f).

### Nonlinear properties of hybridized interlayer excitons

**Fig. 3:**
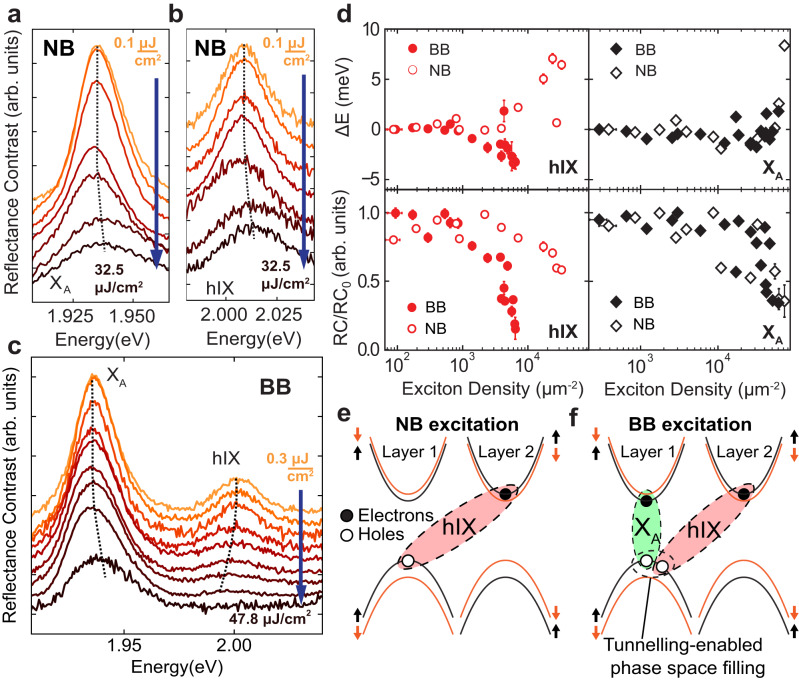
Exciton nonlinearity in MoS_2_ bilayers. RC spectra measured with the NB (FWHM=28 nm) excitation for the X_*A*_ (**a**) and hIX (**b**), and with the BB (FWHM = 50 nm) excitation (**c**) at different fluences. The dashed curves are guide for the eye. **d** The energy shift Δ*E* (top) and normalized integrated RC intensity (bottom) as a function of the exciton density for the hIX (left) and X_*A*_ (right). Solid (open) symbols show the results for the BB (NB) excitation. For the normalized RC we divide the spectrally integrated RC at each laser fluence by its maximum value measured for the whole power dependence. Schematic diagram showing exciton generation under the NB (**e**) and BB (**f**) excitation. In (**e**) only generation of hIX is shown. In (**f**), the holes of the two excitonic species share the same valence band.

We investigate the nonlinear response of X_A_ and hIX in the bare BL flake performing fluence-dependent reflectivity experiments, illuminating the sample with ultrashort (≈150 fs) pump pulses (in a single beam experiment) in both narrow band (NB, full-width at half maximum, FWHM = 28 nm) and broad-band (BB, FWHM = 50 nm) configurations (see “Methods”). Our separate resonant pump-probe experiments have confirmed that the lifetimes of the hIX and X_*A*_ states are considerably longer than the pulse duration of ≈ 150 fs (Supplementary Note S3). Measured RC spectra are shown in Fig. 3a, b for the NB and in Fig. 3c for BB excitation. In the NB case, the excitation was tuned to excite either X_*A*_ or hIX independently, while in the BB case, both resonances were excited simultaneously.

As seen in Fig. 3a, b both X_A_ and hIX spectra behave similarly upon increasing the power of the NB excitation: a blueshift of several meV is observed, accompanied by the peak broadening and bleaching. For the BB excitation, however, a different nonlinear behaviour is observed as shown in Fig. 3c: the broadening and complete suppression of the hIX peak is observed at much lower powers, accompanied by a redshift. This is in contrast to X_A_, whose behaviour is similar under the two excitation regimes.

The resulting energy shifts and intensities are shown in Fig. 3d as a function of the exciton density (see details in Supplementary Note S4 and S5). Figure 3d quantifies the trends observed in Fig. 3a–c showing for the BB excitation an abrupt bleaching of the hIX peak above the hIX density 5 × 10^3^ μm^−2^ accompanied by a redshift of ≈ 4 meV and a 12 meV broadening (see Supplementary Note S6). For the NB case, a similar decrease in peak intensity is observed only around 4 × 10^4^ μm^−2^, accompanied with a peak blueshift of ≈ 7 meV and a broadening exceeding 15 meV (see Supplementary Figure S10). However, it is apparent that the observed behaviour under the two excitation regimes is similar for X_A_. A similar blueshift, broadening and saturation are observed at slightly higher densities compared to the hIX under the NB excitation (Supplementary Note S7). We also find that due to the increased excitonic Bohr radius, the onset of the nonlinear behaviour for X_A_ in bilayers occurs at a lower exciton density than for X_A_ in monolayers (Supplementary Note S8).

We develop a microscopic model to describe the contrasting phenomena under the NB and BB excitation. Under the NB excitation, either X_A_ or hIX excitons are created as sketched in Fig. 3e. In this case, nonlinearity arises from Coulomb exciton-exciton interactions causing the blueshift and dephasing^36^. For simplicity, in the main text we will use a Coulomb potential *V*_Coul_ combining the exchange and direct terms further detailed in Supplementary Note S9. We confirm that for the intralayer exciton-exciton interaction (X_A_-X_A_) the dominant nonlinear contribution comes from the Coulomb exchange processes, as in the monolayer case^36,37^, while for the hIX-hIX scattering the dominant contribution is from the direct Coulomb (dipole-dipole) interaction terms^22^. We find that for the modest electron-hole separation *d* = 0.55 nm in the bilayer, *V*_Coul_ is overall 2.3 times stronger for hIX compared with X_A_. For X_A_ and hIX of the same dipole orientation the Coulomb interaction is repulsive, corresponding to the experimentally observed blueshifts. At the same time, opposite dipole orientation leads to the negative energy shifts, and the total contribution of interlayer scattering depends on possible asymmetry in the system (Supplementary Note 11).

Analysing the shapes of the reflectance spectra in the NB case, we note that they depend on the rates of radiative (Γ_R_) and non-radiative (Γ_NR_) processes. The area under RC curves is described by the ratio Γ_R_/(Γ_R_ + Γ_NR_). This ratio changes under the increased excitation if the rates depend on the exciton densities. Specifically, we account for the scattering-induced non-radiative processes that microscopically scale as Γ_NR_ ∝ ∣*V*_Coul_∣^2^*n*, i.e. depend on the absolute value of the combined matrix elements for the Coulomb interactions and the exciton density *n*^36^. This process allows reproducing the RC behaviour and bleaching at increasing pump intensity. Moreover, it explains stronger nonlinearity for X_A_ in bilayers compared to monolayers. Namely, the scattering scales with the exciton Bohr radius, *V*_Coul_ ∝ *α*, which is larger in the bilayers due to the enhanced screening (Supplementary Note S9). We note that the full analysis of the observed behaviour should take into account the inhomogeneous broadening, the effect of which nonetheless can be neglected for the qualitative analysis presented in the paper.

In the BB case, both X_*A*_ and hIX excitons are generated simultaneously, and together with intraspecies scattering (X_*A*_-X_*A*_ and hIX-hIX), interspecies scattering (X_*A*_-hIX) occurs, similarly to the direct-indirect exciton Coulomb scattering in double quantum wells^38^. Since X_*A*_ and hIX are formed by the holes from the same valence band (Fig. 3f), an additional contribution arises from the phase space filling, i.e. the commutation relations for the excitons (composite bosons) start to deviate from the ideal weak-density limit once more particles are created^39^. For particles of the same flavour, the phase space filling enables nonlinear saturation effects in the strong coupling regime, similar to polariton saturation observed in Ref. ^18^. However, in the presence of several exciton species, we reveal a distinct mixture of the phase space filling and inter-exciton interactions which we term the *tunnelling-enabled nonlinearity*. Specifically, we note that the commutator of the X_A_ annihilation operator ($\hat{X}$) and hIX creation operator (${\hat{I}}^{{{{\dagger}}} }$) is non-zero, $[\hat{X}({{{{{{{\bf{p}}}}}}}}),{\hat{I}}^{{{{\dagger}}} }({{{{{{{\bf{q}}}}}}}})]=-{\hat{B}}_{{{{{{{{\bf{p}}}}}}}},{{{{{{{\bf{q}}}}}}}}}$, meaning that modes are not independent. Here **p**, **q** are exciton momenta and ${\hat{B}}_{{{{{{{{\bf{p}}}}}}}},{{{{{{{\bf{q}}}}}}}}}$ is an operator denoting the deviation from the ideal commuting case (${\hat{B}}_{{{{{{{{\bf{p}}}}}}}},{{{{{{{\bf{q}}}}}}}}}=0$) of distinct bosons where holes do not compete for the valence band space.

This statistical property of modes that share a hole, and experience phase space filling, has consequences for the nonlinear response. Namely, the total energy is evaluated as an expectation value over a many-body state with both X_A_ and hIX excitons, $\left|{N}_{{{{{{{{\rm{X}}}}}}}}},{N}_{{{{{{{{\rm{hIX}}}}}}}}}\right\rangle :=(\mathop{\prod }\nolimits_{{{{{{{{\bf{p}}}}}}}}}^{{N}_{{{{{{{{\rm{X}}}}}}}}}}{\hat{X}}^{{{{\dagger}}} })(\mathop{\prod }\nolimits_{{{{{{{{\bf{q}}}}}}}}}^{{N}_{{{{{{{{\rm{hIX}}}}}}}}}}{\hat{I}}^{{{{\dagger}}} })\left|{\Omega }_{0}\right\rangle$, where *N*_X_ and *N*_hIX_ particles are created from the ground state $\left|{\Omega }_{0}\right\rangle$. If the excitonic modes are independent, the contributions from X_A_ and hIX simply add up. However, the hole coexistence in the valence band induces the excitonic interspecies scattering. The phase space filling then affects both Coulomb-related processes, radiative lifetime of excitonic modes, and scattering amplitudes, which can lead to redshifts for hIX (see Supplementary Notes S9–S11). The interplay of these effects is unique to bilayers as tunnelling is absent in monolayers. Moreover, in contrast to standard epitaxially-grown double quantum wells where the layers are separated up to 10 nm, much smaller interlayer separation in TMD bilayers (0.5 nm) allows for stronger tunnelling-enabled nonlinearity.

We note that the magnitude of shifts depends on the mode populations as well as interaction constants. For instance, the redshift for X_A_ scales as $\Delta {E}_{{{{{{{{{\rm{X}}}}}}}}}_{{{{{{{{\rm{A}}}}}}}}}}={g}_{{{{{{{{\rm{D}}}}}}}}-{{{{{{{\rm{I}}}}}}}}}{n}_{{{{{{{{\rm{hIX}}}}}}}}}$. The population of hIX is much smaller than X_A_ for a given power and the imbalance grows further as the fluence is increased (see Supplementary Note S12). This can explain that the redshift induced by tunnelling-enabled nonlinear processes for X_A_ is less apparent than for hIX under BB illumination. At the same time, the shifts for hIX are proportional to the density of intralayer excitons, ${n}_{{{{{{{{{\rm{X}}}}}}}}}_{{{{{{{{\rm{A}}}}}}}}}}$, and thus the effect of the BB excitation shall be most pronounced for the interlayer mode. In addition, hIX-hIX scattering plays a significant role for increasing nonradiative rates. In this case, interactions of both repulsive and attractive sign lead to enhanced scattering channels (irrespective of dipole orientation), and thus results into RC spectra bleaching at lower hIX exciton densities (Supplementary Note S9). Using the estimated tunnelling-enabled nonlinearity coefficients, we model the RC in the BB regime and qualitatively reproduce the strong bleaching and redshift for hIX at the increased density (see Supplementary Fig. S13).

We observe the same BB-enhanced tunnelling-enabled nonlinearity in samples with lower inhomogeneous broadening (Supplementary Note S13), confirming that our findings are not masked by disorder. In our analysis on the excitonic nonlinearities, we neglect the effect of trions and biexcitons^40,41^, since the natural doping is relatively small in our sample (see Fig. 1c, e) and the excitation densities required to generate a substantial population of biexcitons are considerably higher than those used in our experiments^42–44^. Moreover, we observe a redshift only for interlayer excitons and specifically under broadband excitation, while an excess of charges would have produced the same effect on both intra and interlayer excitons. In fact, the interspecies interactions in our study are mediated through charge tunnelling and cannot be explained as a consequence of the presence of many-body complexes.

### Nonlinear behaviour of dipolar polaritons

**Fig. 4:**
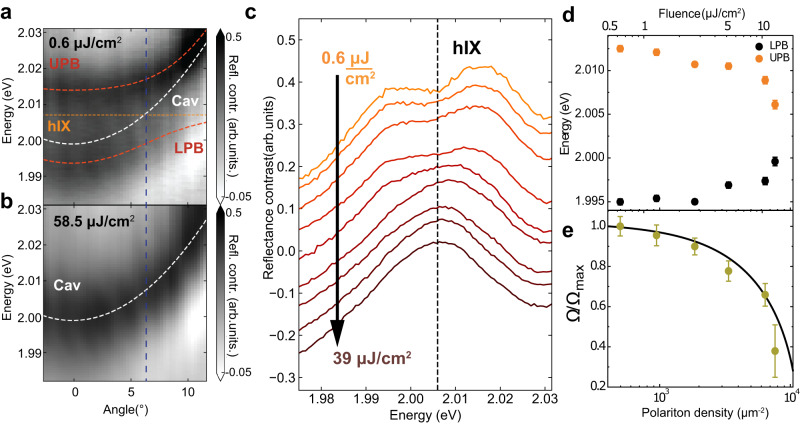
Nonlinear behaviour of dipolaritons. **a**, **b** Reflectance contrast spectra measured at different laser fluences for the MoS_2_ bilayer placed in a monolithic cavity. **a** The low fluence case (0.6 μJ cm^−2^). A clear anticrossing at 6.5^∘^ is observed. Dashed red lines show the results of the fitting using a coupled oscillator model, with two polariton branches LPB and UPB formed. White and orange lines show the energies of the uncoupled cavity mode and hIX state, respectively. The vertical line marks the anticrossing angle. **b** The high fluence case (58.5 μJ cm^−2^). A complete collapse of the strong coupling regime is observed, with the disappearance of the anticrossing and transition into the weak coupling regime. **c** RC spectra measured at the anticrossing at 6.5^∘^ as a function of the laser fluence. **d** Measured UPB and LPB peak energies at 6.5^∘^ as a function of the laser fluence (see top axis) and the corresponding polariton density (bottom axis). **e** Symbols show the Rabi splittings normalized by the Rabi splitting measured at the lowest power (Ω/Ω_*max*_) as deduced from (**d**). The line shows the fitting using our theoretical model (Supplementary Note S9).

We investigate nonlinear properties of dipolar polaritons in a monolithic (fixed-length) cavity created by a silver mirror on top of a PMMA spacer (245 nm thick) covering the hBN-encapsulated MoS_2_ homobilayer placed on the DBR. The cavity mode energy can be tuned by varying the angle of observation (0 degrees corresponds to normal incidence). We use a microscopy setup optimized for Fourier-plane imaging, thus allowing simultaneous detection of reflectivity spectra in a range of angles as shown in (Fig. 4a) displaying the measured polariton dispersion. In this experiment, the cavity mode is tuned around hIX and only two polariton branches LPB and UPB are observed at low fluence of 0.6 *μ*J cm^−2^ with a characteristic Rabi splitting of 17.5 meV. In Fig. 4b, at an increased fluence of 58.5 μJ cm^−2^, only a weakly coupled cavity mode is visible.

Figure 4c shows RC spectra taken at ~6.5^∘^ around the anticrossing at different laser fluences. The collapse of the two polariton peaks into one peak signifying the transition to the weak coupling regime is observed above 25 μJ cm^−2^. The LPB and UPB energies extracted using the coupled oscillator model (Supplementary Fig. S5) are shown in (Fig. 4d). As the polariton density is increased, the LPB and UPB approach each other almost symmetrically, converging to the exciton energy. The corresponding normalized Rabi splitting (Ω/Ω_*max*_, where Ω_*max*_ is measured at low fluence) is  shown in Fig. 4d, e as a function of the total polariton density.

In this experiment, the cavity mode is considerably above the X_*A*_ energy, which therefore is not coupled to the cavity. Hence, the extracted Rabi splittings are fitted with a theoretically predicted trend of Ω for the NB excitation regime (Supplementary Note S9). A nonlinear polariton coefficient *β* = 0.86 *μ*eV*μ*m^2^ is extracted by differentiating the fitted function with respect to the polariton density. Comparing our results to X_A_ intralayer-exciton-polaritons in monolayers in similar cavities^11^, we observe that the nonlinearity coefficient for dipolar interlayer polaritons is about an order of magnitude larger. This is in a good agreement with the theoretically predicted intrinsic nonlinearity of hybridized interlayer polaritons (Supplementary Note S9), and with our experimental data for X_A_ taken on a monolayer embedded in a microcavity (Supplementary Note S14).

## Discussion

In summary, we report the nonlinear exciton and exciton-polariton behaviour in MoS_2_ homobilayers, a unique system where hybridized interlayer exciton states can be realized having a large oscillator strength. We find that nonlinearity in MoS_2_ bilayers can be enhanced when both the intralayer and interlayer states are excited simultaneously, the regime that qualitatively changes the exciton-exciton interaction through the tunnelling-enabled nonlinearity. This approach enriches the exciton nonlinear behaviour in atomically thin semiconductors, leading to unique effects compared to any other semiconducting system. In this broad-band excitation regime, the bleaching of the hIX absorption occurs at 8 times lower hIX densities compared to the narrow-band excitation case when the interlayer excitons are generated on their own. In addition to this, we find that the dipolar nature of hIX states in MoS_2_ homobilayers already results in 10 times stronger nonlinearity compared with the intralayer excitons in MoS_2_ monolayers. Thus, we report on an overall enhancement of the exciton nonlinearity by nearly two orders of magnitude.

Thanks to the large oscillator strength, hIX can enter the strong coupling regime in MoS_2_ bilayers placed in microcavities, as realized in our work. Similarly to hIX states themselves, dipolar polaritons also show 10 times stronger nonlinearity compared with exciton-polaritons in MoS_2_ monolayers. We conclude that dipolaritons in MoS_2_ bilayers uniquely combine strong nonlinearity, large Rabi splitting and fabrication reproducibility among other TMD systems studied so far^9,11,18^ (see Supplementary Note 14). We note that in the monolayer samples, the nonlinearity arising from Pauli blocking is only pronounced in the strong light-matter coupling regime^18,45^, and is not significant in the weak coupling. On the contrary, for bilayers we discover other nonlinear processes that crucially arise due to the presence of interlayer tunnelling. This is specific to the broad band (BB) illumination regime, where the simultaneous occupation of intra- and interlayer exciton states effectively activates this channel of nonlinearity. We expect that in microcavities where the cavity mode is coupled to both hIX and X_*A*_ in MoS_2_ bilayers, and the excitation similar to the broad-band regime can thus be realized, the nonlinear polariton coefficient will be dramatically enhanced owing to the tunnelling-enabled nonlinearity effect, allowing highly nonlinear polariton system to be realized. We thus predict that MoS_2_ bilayers will be an attractive platform for realization of quantum-correlated polaritons with applications in polariton logic networks^46^ and polariton blockade^47,48^.

After the submission of this manuscript another article reporting strong coupling and nonlinear effects in MoS_2_ bilayers was published^49^.

## Methods

### Sample fabrication

The hBN/MoS_2_/hBN heterostructures were assembled using a PDMS polymer stamp method. The PMMA spacer for the monolithic cavity was deposited using a spin-coating technique, while a silver mirror of 45 nm was thermally evaporated on top of it.

### Optical studies

Broad-band excitation was used to measure the reflectance contrast (RC) spectra of the devices at cryogenic temperatures, defined as RC = (*R*_sub_ − *R*_BL_)/*R*_sub_, where *R*_sub_ and *R*_BL_ are the substrate and MoS_2_ bilayer reflectivity, respectively. For the magnetic field studies the same RC measurements were performed using unpolarized light in excitation and with *λ*/4 and *λ*/2 waveplates and a linear polarizer in collection to resolve *σ*^+^ and *σ*^−^ polarized light. The low temperature measurements using the tunable cavity were carried out in a liquid helium bath cryostat (T = 4.2K) equipped with a superconducting magnet and free beam optical access. We used a white light LED as a source. RC spectra were measured at each cavity length and were integrated over the angles within 5 degrees from normal incidence. The RC spectra measured in the cavity are fitted using Lorentzians. The peak positions are then used to fit to a coupled oscillator model, producing the Rabi splitting and the exciton and cavity mode energies.

The measurements on the monolithic cavity were performed in a helium flow cryostat (T = 6K). For the power-dependent RC experiments, we used supercontinuum radiation produced by 100 fs Ti:Sapphire laser pulses at 2 kHz repetition rate at 1.55 eV propagating through a thin sapphire crystal. The supercontinuun radiation was then filtered to produce the desired narrow-band excitation.

### Density calculations

All the exciton and polariton densities were calculated following the procedure introduced by L. Zhang et al.^11^, taking into account the spectral overlap of the spectrum of the excitation laser and the investigated exciton peak (see further details in Supplementary Note S4).

## Supplementary information

Supplementary Information

Peer Review File

## Data Availability

The data that support the findings of this study are available in the MARVEL public repository (MARVEL Materials Cloud Archive: https://archive.materialscloud.org) with the same title as this paper.
