# Supplementary material for: Interspecies exciton interactions lead to enhanced nonlinearity of dipolar excitons and polaritons in MoS2 homobilayers
Source: Nat Commun. 2023 Jun 27;14:3818. doi: 10.1038/s41467-023-39358-9 (PMC10300037; doi:10.1038/s41467-023-39358-9)
Supplement: Supplementary file 1 — Supplementary Information [file 41467_2023_39358_MOESM1_ESM.pdf]

Supplementary Information: Interspecies exciton interactions lead to enhanced nonlinearity of dipolar excitons and polaritons in MoS_2 bilayers

Charalambos Louca,[1,*] Armando Genco,[2,†] Salvatore Chiavazzo,[3] Thomas P. Lyons,[1,4] Sam Randerson,[1] Chiara Trovatello,[2] Peter Claronino,[1,5] Rahul Jayaprakash,[1] Xuerong Hu,[1] James Howarth,[6,7] Kenji Watanabe,[8] Takashi Taniguchi,[8] Stefano Dal Conte,[2] Roman Gorbachev,[6,7] David G. Lidzey,[1] Giulio Cerullo,[2] Oleksandr Kyriienko,[3] and Alexander I. Tartakovskii[1,‡]

[1]*Department of Physics and Astronomy, The University of Sheffield, Sheffield S3 7RH, UK*

[2]*Dipartimento di Fisica, Politecnico di Milano, Piazza Leonardo da Vinci, 32, Milano, 20133, Italy*

[3]*Department of Physics, University of Exeter, Stocker Road, Exeter, EX4 4PY, UK*

[4]*RIKEN Center for Emergent Matter Science, Wako, Saitama, 351-0198, Japan*

[5]*Department of Physics and Mathematics, University of Hull, Rober Blackburn, Hull HU6 7RX, UK*

[6]*National Graphene Institute, University of Manchester, Manchester, UK*

[7]*Department of Physics and Astronomy, University of Manchester, Manchester, UK*

[8]*Advanced Materials Laboratory, National Institute for Materials Science, 1-1 Namiki, Tsukuba, 305-0044, Japan*

In this Supplementary Information we include Notes discussing experimental details of the observed nonlinear behaviour of polaritons and excitons and in MoS_2 homobilayers, as well as providing a theoretical explanation of nonlinear processes. Each Note starts from a new page, and targets a specific aspect in our analysis.

SUPPLEMENTARY NOTE S1: THEORETICAL ESTIMATE OF EXCITON PROPERTIES — ENERGY AND HYBRIDISATION

In this Supplemental Note we describe details of the theoretical description of MoS_2 homobilayers. Properties of excitons in a bilayer system are discussed in the main text, and we support them introducing a corresponding theoretical model. The optical response of the system is characterised mainly by the response of three different species of quasi-particles, being X_A, hIX and hX_B excitons (see main text). However, we keep our analysis generic, and in this section we describe the system in

terms of original (non-hybridised) modes corresponding to X_A, X_B, IX_B, and IX_A. In the main text, IX_B is referred as the IX mode, as it represents a dominant interlayer mode. We also take into account for the system's symmetry explicitly. Here we provide a physical description of the homobilayer physics and estimate the system parameters. In particular, we estimate exciton Bohr radii and a hole tunnelling rate, which are relevant for investigating nonlinear properties of homobilayer samples.

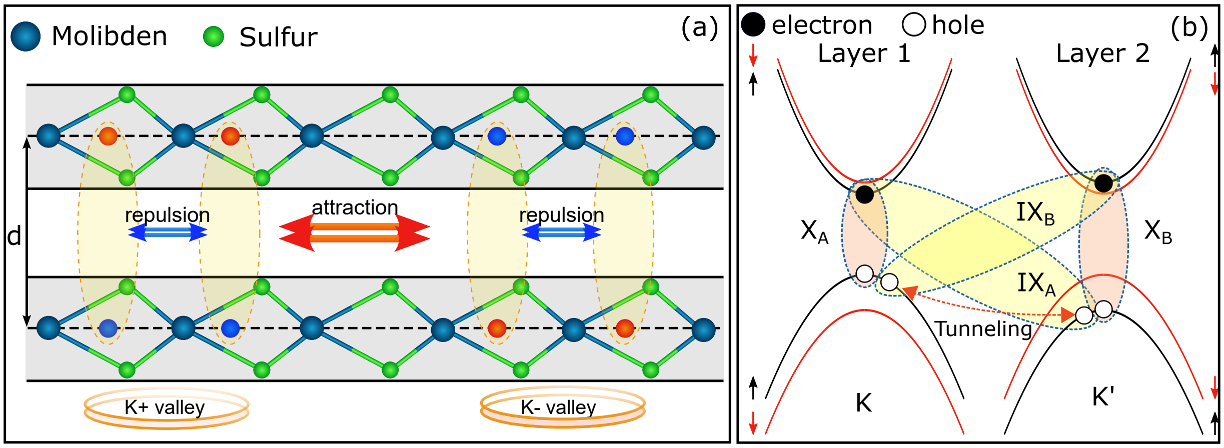

Supplementary Figure S1. (a) **Side view of 2H-stacked MoS_2 bilayer.** Blue spheres are Mo (molybdenum) atoms and green are S (sulphide) atoms. Electrons (light blue spheres) and holes (red spheres) are represented in the picture, and we show the real space scheme with excitons in separate valleys. With no symmetry breaking, IX excitons with same orientation repel via Coulomb interaction, while excitons with opposite orientation attract each other. In absence of external fields, the centre of charge is located in between the sulfure planes [1]. (b) **Band structure of a homobilayer with interlayer tunnelling.** The schematic accounts for the hole tunnelling, and shows relevant excitonic modes. With holes being hybridized, intralayer excitons X (A- or B-type, labelled by corresponding indices) and couple to interlayer excitons IX (A- or B-type, respectively). This leads to hybrid indirect excitons that can be excited with a broadband pump.

We consider a MoS_2 homobilayer system with 2H stacking (see Fig. **S1**). This is comprised of two parallel layers of MoS_2, with centres located at a distance d from each other (charge separation distance). The physics of bilayers is defined by properties of electrons and holes that interact through the Keldysh-Rytova potential [2–4], being different for in-plane and out-of-plane interaction [5]. Within the $k \cdot p$ framework, electrons and holes are treated as particles with effective mass provided by a band dispersion. In MoS_2 the typical values for effective masses are 0.42 m_e

for conduction bands and 0.55 m_e for the valence bands (with m_e being the free electron mass) [6, 7]. The attractive Kledysh-Rytova potential has a different form depending on the relative position between particles. We call $V_{\mathrm{KR}}^{\mathrm{intra}}$ the attractive potential of particles being in the same layer, and $V_{\mathrm{KR}}^{\mathrm{inter}}$ the attractive potential of particles being in separate layers. In momentum space the different potentials read as

$$V_{\mathrm{KR}}^{\mathrm{intra}}(\mathbf{q}) = -\frac{e^2}{4\pi\epsilon\epsilon_0}\frac{1+(r_0q/\epsilon)(1-\exp(-2qd))}{(1+r_0q/\epsilon)^2-(r_0q/\epsilon)^2\exp(-2qd)}, \tag{S1a}$$

$$V_{\mathrm{KR}}^{\mathrm{inter}}(\mathbf{q}) = -\frac{e^2}{4\pi\epsilon\epsilon_0}\frac{\exp(-qd)}{(1+r_0q/\epsilon)^2-(r_0q/\epsilon)^2\exp(-2qd)}, \tag{S1b}$$

where e is the electron charge, ϵ_0 is the vacuum permittivity, ϵ in an average environment permittivity, r_0 is a screening length (defined as for monolayers), and $\mathbf{q}$ is an exchanged particle momentum [5]. We compute a binding energy of an exciton bound state by assuming the Keldysh-Rytova attractive potential and free particle dispersion defined by the effective masses. The relative motion of an exciton can be described by the ansatz wave function $\phi(\rho) = \sqrt{2/\pi\alpha^2}\exp(-\rho/\alpha)$, with ρ being the in-plane projection of electron-hole distance and α the exciton Bohr radius. This describes well an internal structure of an exciton, and gives the information about its shape, collected in the Bohr radius. In Fourier space, the function $\phi(q)$ reads

$$\phi(q) = \sqrt{\frac{2}{\pi}}\frac{\alpha}{(1+q^2\alpha^2)^{3/2}}. \tag{S2}$$

We use this ansatz function [Eq. (S2)] and interaction potentials [Eqs. (S1)] to find the excitons binding energy via variational procedure by minimizing the Bohr radius α.

In the analysis, we consider a fully symmetrized system where excitons hybridize via the hole tunnelling [see Fig. **S1**], and introduce two effective coupling constants $J_{\mathrm{A,B}}$, mediating the coupling between $\mathrm{X}_{\mathrm{A,B}}$ and $\mathrm{IX}_{\mathrm{A,B}}$. The hybridization between

modes follows from the effective Hamiltonian based on coupled damped oscillators. Specifically, the Hamiltonian H_{hIX} of a coupled $\mathrm{X_B}$-$\mathrm{IX_B}$ system can be written as a two-by-two matrix, which reads

$$H_{\mathrm{hIX}} = \begin{pmatrix} E_{\mathrm{X_B}} - i\Gamma_{\mathrm{X_B}} & J_{\mathrm{B}} \\ J_{\mathrm{B}} & E_{\mathrm{IX_B}} - i\Gamma_{\mathrm{IX_B}} \end{pmatrix}. \tag{S3}$$

Here $E_{\mathrm{X_B,IX_B}}$ are exciton energies, and introduce broadening for the two exciton modes $\Gamma_{\mathrm{X_B,IX_B}}$, which in general combine radiative and nonradiative losses for the specified mode. Similarly, we can write the coupling between $\mathrm{X_B}$-$\mathrm{IX_B}$, simply swapping indices from B to A.

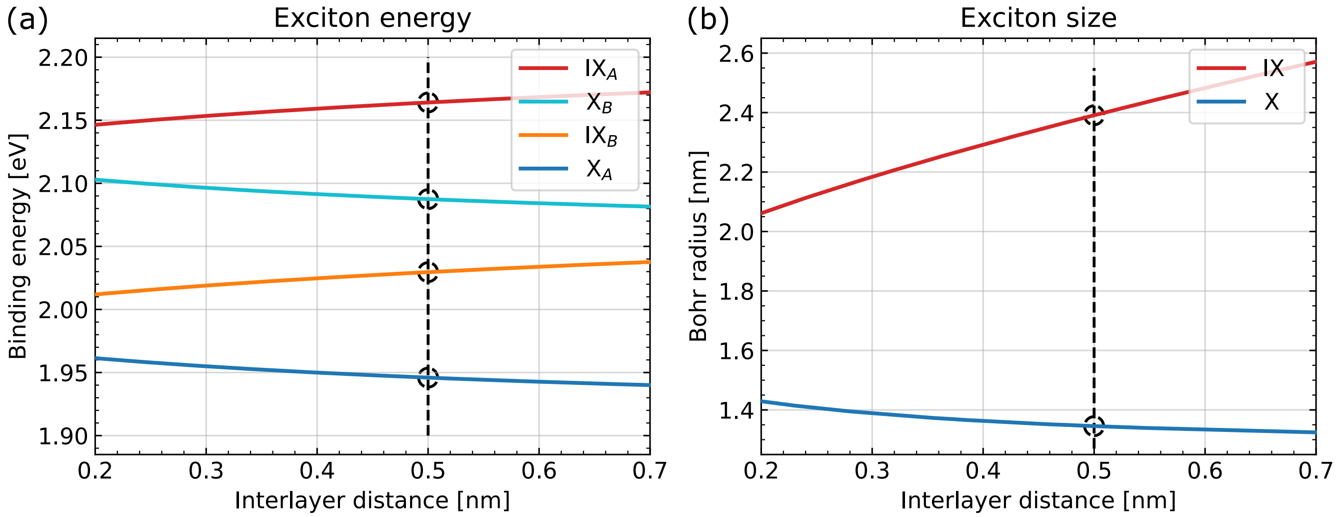

Supplementary Figure S2. **Exciton properties as a function of interlayer distance.** **(a)** Energy of excitons for the varying d. Separate curves show the evolution of $\mathrm{IX_A}$ (red curve), $\mathrm{X_B}$ (cyan curve), $\mathrm{IX_B}$ (orange curve), and $\mathrm{X_A}$ (blue curve). The redshift of intralayer excitonic modes with the increase of interlayer distance is due to reduced screening effects, being characteristic to the Keldysh-Rytova potential, Eq. (S1). On the contrary, the IX modes experience blueshifts. This is due to the lower attraction of particles located in separate layers. The black dashed line is the distance chosen to match energies to the experimental data. **(b)** Dependence of Bohr radius on the interlayer distance. Here, we only present one curve for intralayer and interlayer excitons as both A and B excitons exhibit the degenerate behaviour (due to similar masses). The blue curve corresponds to the Bohr radius for direct excitons, and the red curve is for the indirect excitons. Both plots (a) and (b) show similar increase (or decrease) with distance d as the screening mechanism affects the considered parameters the same way. This analysis reveals the IX Bohr radius to be roughly twice as much as the X one.

Given the significant tunnelling rate, the system can be described in terms of hybrid excitonic states, being mixtures of X and IX modes. This leads to a significant

out-of-plane dipole moment and enhanced light-matter interaction for the hybrid modes. Consequently, energy levels split due to tunnelling according to relation $\Delta_{\mathrm{hIX}} = \sqrt{4J_{\mathrm{B}}^2 + (E_{\mathrm{X_B}} - E_{\mathrm{IX_B}})^2 - (\Gamma_{\mathrm{X_B}} - \Gamma_{\mathrm{IX_B}})^2}$. Accordingly, measured energies are affected by the hole tunnelling and broadening.

In our analysis, we compute the binding energies of the four excitonic species, namely $\mathrm{X_A}$, $\mathrm{X_B}$, $\mathrm{IX_A}$ and $\mathrm{IX_B}$. In Fig. **S2**(a) and (b) we plot respectively the binding energy and Bohr radius of separate excitonic species as a function of interlayer distance d. In our analysis, we considered a band gap of 2.12 eV, and spin-orbit splitting of 3.5 meV for conduction band and 138 meV for valence band, as indicated by ab initial calculations [7]. Finally, by comparing with experimental measurements, we conclude the interlayer distance is $d = 0.55$ nm, with tunnelling energy being $J_{\mathrm{B}} = 46$ meV. Consequently, the hybridization of B modes is such that 23% of $\mathrm{X_B}$ oscillator strength is transferred to $\mathrm{IX_B}$, in agreement with previous observations [6]. Note that this estimates are performed in the absence of the strong light-matter coupling regime, where additional mode hybridization occurs, leading to an additional distribution of the dipole moment.

Finally, in Fig. **S2**(b) we show the evolution of particle Bohr radii with the interlayer distance. We respectively call the Bohr radius of direct and indirect exciton α_{D} and α_{I}. The blue curve is the Bohr radius of X, while the orange one described the IX modes. With $\mathrm{X_A}$ and $\mathrm{X_B}$ being very similar, we describe both with one orange curve. The energy separation is provided only by the spin-orbit splitting. Typical values of α_{D} are approximately 1 nm, with α_{I} being approximately 2 nm. Note the opposite behaviour of α_{D} and α_{I} with distance. hIX Bohr radius grows with distance due to the reduced attraction between particles in separate layers. On the contrary, by increasing the interlayer distance, we see the reduced screening for

particles in the same layer, resulting in a decrease of the Bohr radius and consequent increase of the binding energy.

SUPPLEMENTARY NOTE S2: COUPLED OSCILLATOR MODEL

A full picture of our system, corresponding to MoS_2 homobilayer, has to take into account 4 different modes coupling to each other: only IX and X_B hybridise through a tunneling parameter, while X_A and X_B can couple with the cavity due to their high oscillator strength. We can then simplify this picture by rewriting the IX and X_B states in terms of the new basis of hybridised modes, hIX and hX_B, as defined in the main text, all of them now capable of a coupling with the cavity mode. The corresponding Hamiltonian reads

$$H = \begin{pmatrix} E_{\mathrm{c}} & \Omega_{\mathrm{X_A}} & \Omega_{\mathrm{hIX}} & \Omega_{\mathrm{hX_B}} \\ \Omega_{\mathrm{X_A}} & E_{\mathrm{X_A}} & 0 & 0 \\ \Omega_{\mathrm{hIX}} & 0 & E_{\mathrm{hIX}} & 0 \\ \Omega_{\mathrm{hX_B}} & 0 & 0 & E_{\mathrm{hX_B}} \end{pmatrix}, \tag{S4}$$

where E_{c} in the energy of the cavity mode, and $E_{\mathrm{X_A}}$, E_{hIX}, $E_{\mathrm{hX_B}}$ denote energies of the respective excitonic modes. Here, $\Omega_{\mathrm{X_A}}$, Ω_{hIX}, and $\Omega_{\mathrm{hX_B}}$ are corresponding matrix elements for light-matter coupling (Rabi splittings).

Due to the large energy separation between the resonances, each splitting can be fitted to a two level oscillator model independently. In our case, the spectra from the open cavity scans at piezo voltages close to resonant anticrossings between the cavity and either X_A or hIX, were fitted with Lorenzian functions. The results were then fitted to the respective Hamiltonians of two coupled oscillators, such that $H_{\mathrm{A}} = \begin{pmatrix} E_{\mathrm{c}} & \Omega_{\mathrm{X_A}} \\ \Omega_{\mathrm{X_A}} & E_{\mathrm{X_A}} \end{pmatrix}$ and $H_{\mathrm{h_{IX}}} = \begin{pmatrix} E_{\mathrm{c}} & \Omega_{\mathrm{hIX}} \\ \Omega_{\mathrm{hIX}} & E_{\mathrm{hIX}} \end{pmatrix}$ and the values of Rabi splittings and resonant energies were extracted. The results of the fit are shown in Fig. **S3**

In the presence of an out of plane magnetic field of 8 T, the same scans were repeated with unpolarised excitation and detecting opposite circularly polarized

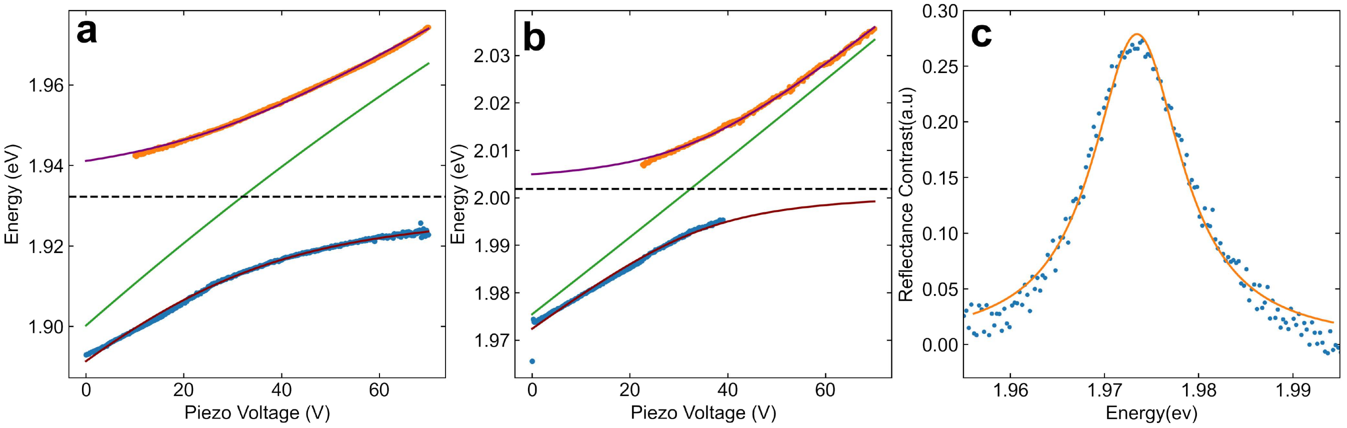

Supplementary Figure S3. **Coupled oscillator model fits. a), b)** Orange and blue dots represent the extracted peak energies of individual spectra near the a) X_A and b) hIX energies. The solid purple (UPB) and red (LPB) curves are the solutions to the fitted coupled oscillator Hamiltonians, and the green solid line is the extracted cavity mode energy as a function of voltage. **c)** shows a spectrum of the uncoupled cavity mode. A Lorenzian peak (orange) is fitted to the data (blue dots) and the linewidth is extracted to be equal to 11 meV, confirming that the condition for strong coupling regime is met, $\Omega^2 > ({\gamma_c}^2 + {\gamma_x}^2)/2$ as stated by [8].

light σ^+/σ^- at each piezo voltage. Fig. **S4** shows the coupled oscillator fits with data from σ^+ and σ^- detection of the X_A scan. It can be seen that the X_A-polaritons exhibit an opposite sign of Zeeman splitting compared to hIX-polaritons (shown in the main text).

Coupled oscillator models were also used for our monolithic cavity sample whose angular dispersion was measured with Fourier space imaging, as mentioned in the main text. The colour map with the extracted data points is presented in Fig. **S5**, with the overlaid couple oscillator fits.

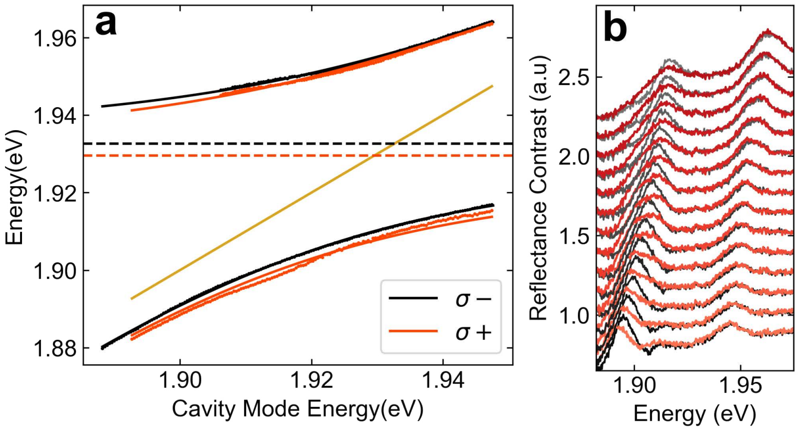

Supplementary Figure S4. **Zeeman Splitting of X_A-polaritons. a)** Tunable cavity RC scans near X_A energies. Orange and black colours represent σ^+ and σ^- detection respectively. Dots represent the extracted peak energies of individual spectra. The solid orange and black curves are the solutions to the fitted coupled oscillator Hamiltonians and the golden solid line is the extracted cavity mode energy. The extracted exciton energies are shown in the plot as the dashed horizontal lines of the corresponding colour. It can be seen that the Zeeman splitting of X_A polaritons is of opposite sign to that of the hIX. The deviation of the σ^+ LPB datapoints from the coupled oscillator model solution (solid line) is due to the presence of the fully polarised trion at $\approx$ 1.91 eV. This can be seen as a broadening due to weak coupling of the lower energy peak of the orange (σ^+) spectra in **b)**, where a cascade plot of the spectra near resonance in the two polarizations is shown.

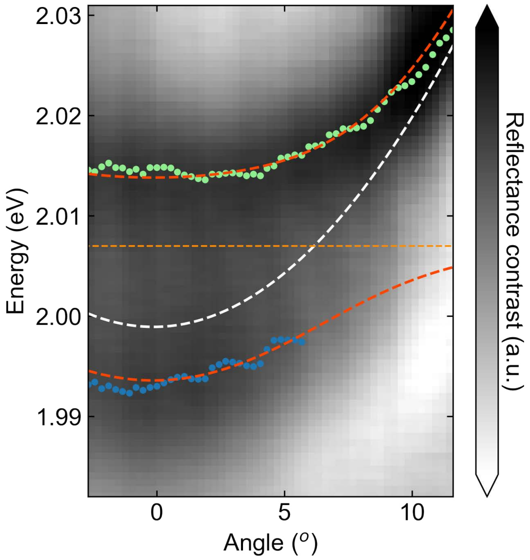

Supplementary Figure S5. **Coupled oscillator model fits on monolithic cavity.** Monolithic cavity dispersion near hIX with couple oscillator model fits. Blue and green points show the extracted peak positions of the LPB and UPB, respectively, from the spectra at each angle. These are then fitted to the two level coupled oscillator model and the solutions LPB and UPB are shown as dark orange dashed curves. The extracted cavity mode and exciton energy are shown as white and light orange dashed curves, respectively.

SUPPLEMENTARY NOTE S3: PUMP-PROBE RESONANT SPECTROSCOPY

We performed time-resolved resonant pump-probe spectroscopy on the encapsulated BL MoS_2 out of the cavity, to measure the X_A and hIX lifetimes in our system. Due to the small size of the samples, a microscopy setup allowing transient reflection measurements at low temperatures has been employed (Fig. **S6** (a)). The setup is powered by an amplified Ti:sapphire laser (Coherent Libra) generating 100 fs pulses at 800 nm (1.55 eV) with 2 mJ pulse energy and 2 kHz repetition rate. A fraction of the laser output is used to seed a non-collinear optical parametric amplifier (NOPA) in the visible energy range. The generated pump pulses are modulated by a mechanical chopper at 500 Hz frequency. The broad-band probe pulses consist of a white-light continuum (WLC), generated from a 1-mm thick sapphire plate pumped by focusing the 800 nm output of the main laser. Pump and probe pulses are synchronized by means of a motorized delay stage. Pump and probe beams are then collinearly combined by a thin dichroic beam splitter and focused on the sample using an objective lens (NA=0.3), resulting in a $4\mu m$ spot size. The samples are placed in a closed-cycle helium cryostat reaching a temperature of 6 K. The spatial overlap of the sample with the pump and probe spots is obtained by a three-axis (xyz) mechanical translation stage coupled to a home-built imaging system. After the interaction with the excited sample, the reflected probe pulse is collimated by the same objective lens and then sent to a spectrometer equipped with an electronically cooled Si CCD, to measure the differential reflection ($\Delta R/R$) signal.

Figure **S6** (b,c) shows the temporal exciton dynamics measured as the absorption bleaching signal in transient reflectivity, taken at the peak wavelengths of the pump-

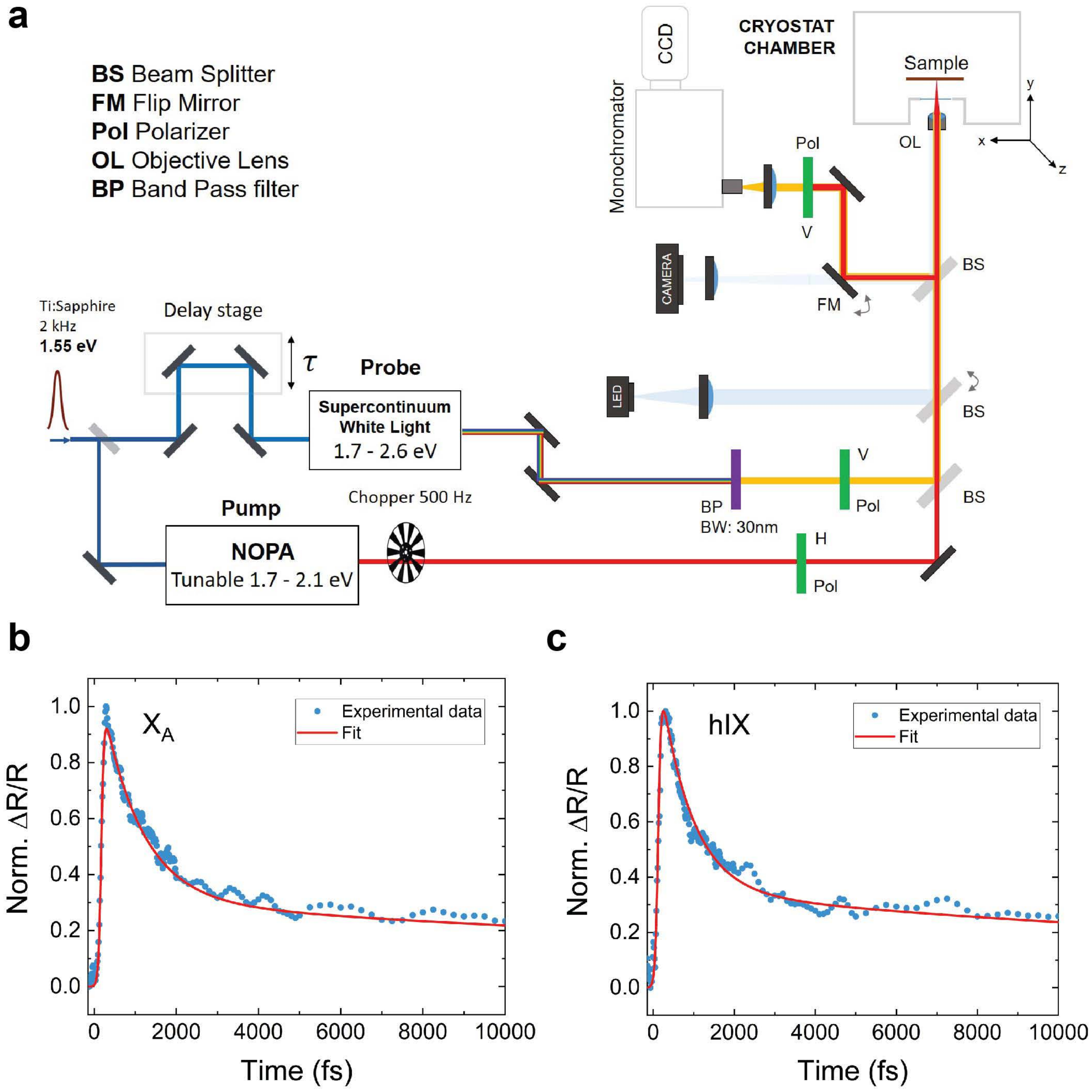

Supplementary Figure S6. **a)** Schematics of the pump-probe microscopy setup used for the experiments on the MoS_2 bilayers. **b, c)** Transient reflectivity traces for X_A (b) and hIX (c) taken at 643 nm and 622 nm respectively. The red curves refer to the fitted bi-exponential decay function.

probe traces, 643 nm and 622 nm for X_A and hIX respectively. For this experiment narrow-band pump pulses (FWHM=10nm) are tuned in resonance with each probed exciton and cross-polarized with respect to the probe pulses. The pump beam is then filtered by an additional polariser placed in the detection path before reaching

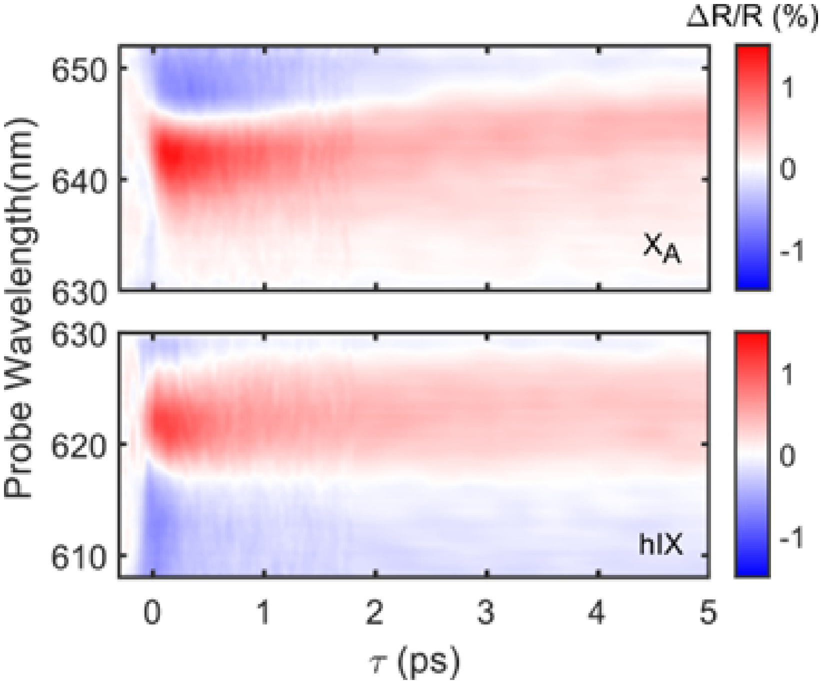

Supplementary Figure S7. Color map of the $\Delta R/R$ signal measured on the encapsulated MoS_2 bilayer at 8K as a function of the delay time τ, probing in the two different spectral windows of X_A and hIX. For these measurements we tuned the peak wavelength of the pump pulses in resonance with each exciton (at 640 nm and 620 nm respectively).

the CCD. The probe spectral window has a narrow bandwidth of 30 nm with a central wavelength fixed at each exciton peak wavelength. The excitons decay traces show a fast component more prominent than the slow one, and can be fitted with a bi-exponential function convoluted with a Gaussian, taking into account the instrument response function. The resulting decay times are $\tau_{\text{fast}} \approx 950$ fs, $\tau_{\text{slow}} \approx 20$ ps for X_A, and $\tau_{\text{fast}} \approx 780$ fs, $\tau_{\text{slow}} \approx 18$ ps for hIX. Considering the degeneracy of pump and probe energies in our experiments, the fast decay can be attributed to electron-phonon scattering processes from the K point to the lowest energy Q point of the Brillouin zone [9, 10], while the slow component is possibly related to radiative [11] or defect-mediated non-radiative recombination [12]. We conclude that in our MoS_2 bilayer sample the fast and slow decay times for both X_A and hIX are much longer than the temporal width of the probe pulses ($\approx$ 150 fs). Figure **S7** shows the full transient reflectivity maps as a function of wavelength and delay

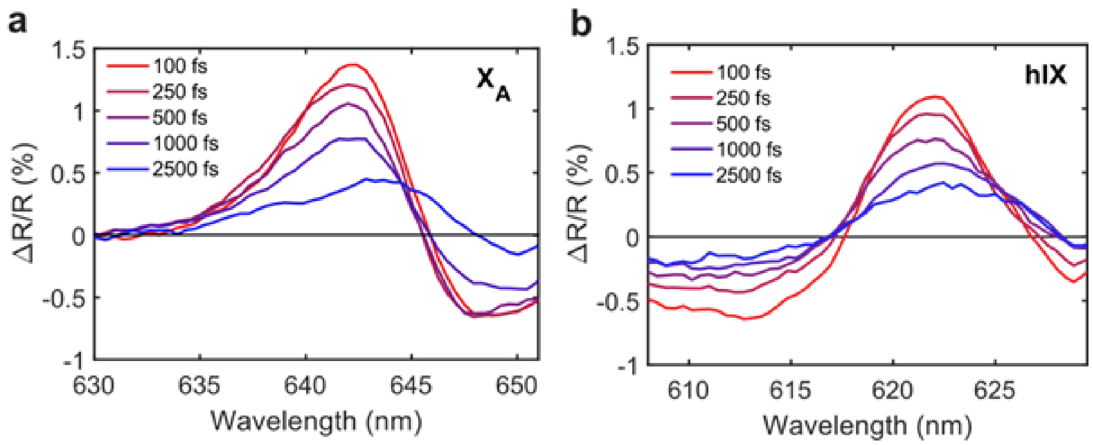

Supplementary Figure S8. Spectral cross-sections of the ΔR/R maps shown in Fig.S7, taken at different delay times, in the spectral windows of the X_A (**a**) and hIX (**b**) excitons of the MoS_2 bilayer.

time, from which the time-resolved traces of Fig. **S6** are extracted. Immediately after time zero, positive and negative signals appear in the pump-probe maps. In these experiments, such features are attributed to pump-induced modification of the excitonic resonance (i.e. reduction of oscillator strength, broadening and shift in energy). The temporal cross-sections of Fig. **S6** are taken at the wavelength of the maximum positive signal for each experiment, which in this case traces the optical saturation of the exciton as a function of time, directly related to the excitonic population density decay. The spectral variations of the exciton optical response are evident in the transient reflectivity spectra plotted in Fig. **S8** for different pump-probe delay times. The prominent positive peaks are mostly a consequence of the pump pulses inducing absorption saturation of the excitonic transitions due to the Pauli blocking effect, [13], while the less intense negative features are generally the result of the renormalization of the exciton energy due to the transient reduction of the Coulomb screening [14]. For both the X_A and hIX, the effect of exciton-exciton interactions can also lead to an energy shift of the excitonic resonance at small time delays, when resonantly pumped [15].

SUPPLEMENTARY NOTE S4: DENSITY ESTIMATION

Exciton and polariton densities were calculated using an experimental approach considering a convolution of the laser profile and the Reflectance Contrast spectra, as in [16]. Reflectance contrast, A_{res}, represents with good approximation the absorption of the each excitonic/polaritonic resonance [16]. Power absorbed by each exciton/polariton, P_{res}, can be calculated as

$$P_{\mathrm{res}} = \frac{P \int L(E)\mathrm{A}_{\mathrm{res}}(E)\mathrm{d}E}{I_{\mathrm{laser}}}, \tag{S5}$$

where P is the experimentally measured power, $\int L(E)\mathrm{A}_{\mathrm{res}}(E)\mathrm{d}E$ is the convolution of the laser spectrum profile, L(E), and the Reflectance Contrast spectrum in the range of energies of the resonant transition peak and $I_{\mathrm{laser}} = \int L(E)\mathrm{d}E$ is the laser spectrum integrated intensity.

The expression (S5) can then be used to calculate the particle density, n_{res}, considering the laser repetition rate, R_{laser}, laser spot size, S_{laser}, and the excitation central energy, E_{res}. Explicitly, an estimate for the density reads

$$n_{\mathrm{res}} = \frac{P_{\mathrm{res}}}{R_{\mathrm{laser}} E_{\mathrm{res}} S_{\mathrm{laser}}}. \tag{S6}$$

We note that for the polariton density estimations, since A_{res} is dependent on the angle, P_{res} was calculated integrating all the spectral quantities in both the energy and angular range of LPB and UPB. As such, the densities calculated with this procedure are the total polariton densities, which consider both LPB and UPB.

For the values of polariton/exciton densities the error, $\epsilon_{\mathrm{n}} = \sqrt{\left(\frac{\epsilon_{\mathrm{P_{res}}}}{\mathrm{P_{res}}}\right)^2 + \left(\frac{\epsilon_{\mathrm{E_{res}}}}{\mathrm{E_{res}}}\right)^2}$, is propagated with respect to standard error analysis rules [17].

SUPPLEMENTARY NOTE S5: HIX AND X_A SPECTRAL FLUENCE DEPENDENCIES

Data shown in Fig. 3 of the main text are presented here in Supplementary Figure **S9** as a function of the raw spectral fluence. We define spectral fluence as the experimentally measured fluence normalised by the illumination spectral width in electron volts.

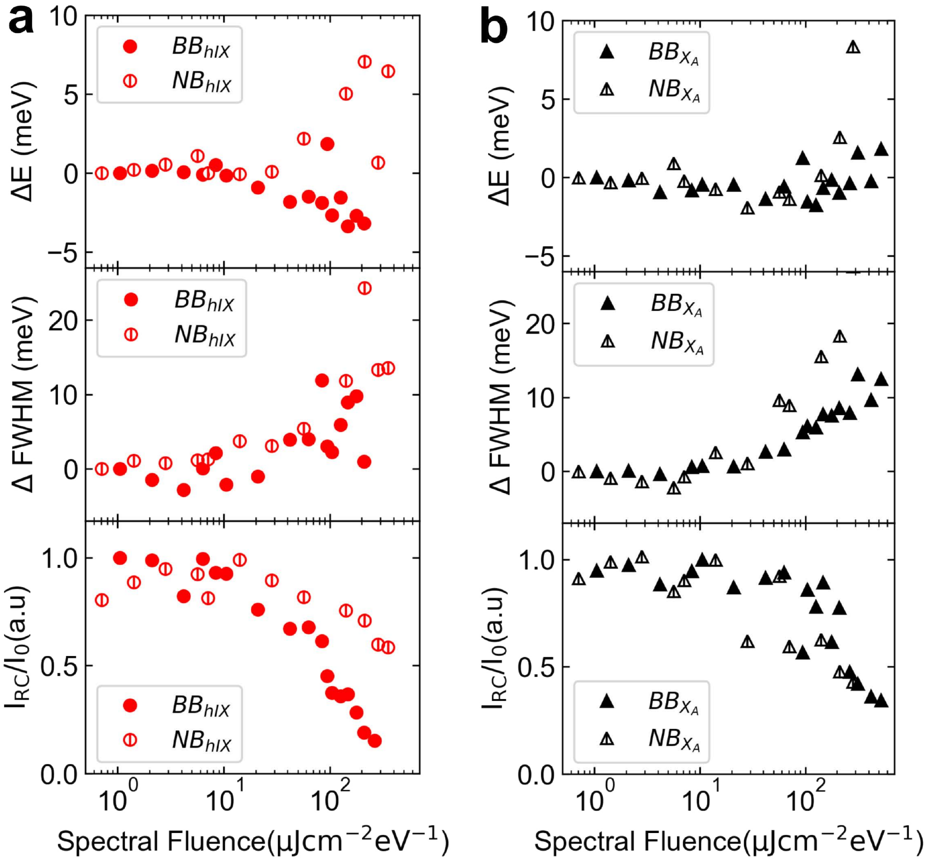

Supplementary Figure S9. **MoS_2 bilayer excitons nonlinear behaviour against spectral fluence a), b)** From top to bottom: plots of the energy shift (ΔE), linewidth variation (ΔFWHM) and normalised integrated intensity (I_{RC}/I_0, where I_0 is the maximum integrated intensity) as a function of the incident fluence normalised by the spectral width (Spectral Fluence) for hIX (red marks in **d**) and X_A (black marks in **e**) peaks measured in RC. Solid marks relate to the experiments with BB excitation, covering both the hIX and the X_A, while open marks refer to the NB excitation, at either the hIX or the X_A energy.

SUPPLEMENTARY NOTE S6: HIX AND X_A FLUENCE-DEPENDENT LINE BROADENING

In Fig. **S10** we shown the density dependence for the broadening of excitonic lines.

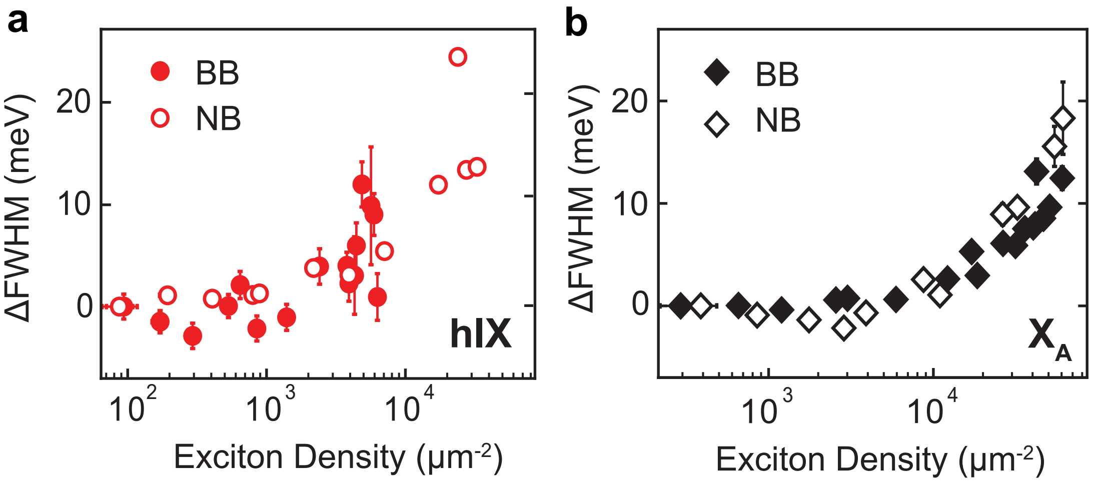

Supplementary Figure S10. Linewidth variation (ΔFWHM) measured in RC for MoS_2 bilayer sample as functions of hIX (a) and X_A (b) densities.

SUPPLEMENTARY NOTE S7: COMPARISON OF HIX AND X_A NONLINEARITY UNDER NB ILLUMINATION

As mentioned in the text, the nonlinear behaviour of hIX is a slightly enhanced compared to X_A. The Supplementary Figure **S11** shows the data of Fig. 3 in the main text such that a direct comparison between the two exciton species under separate narrow band (NB) illumination can be evaluated.

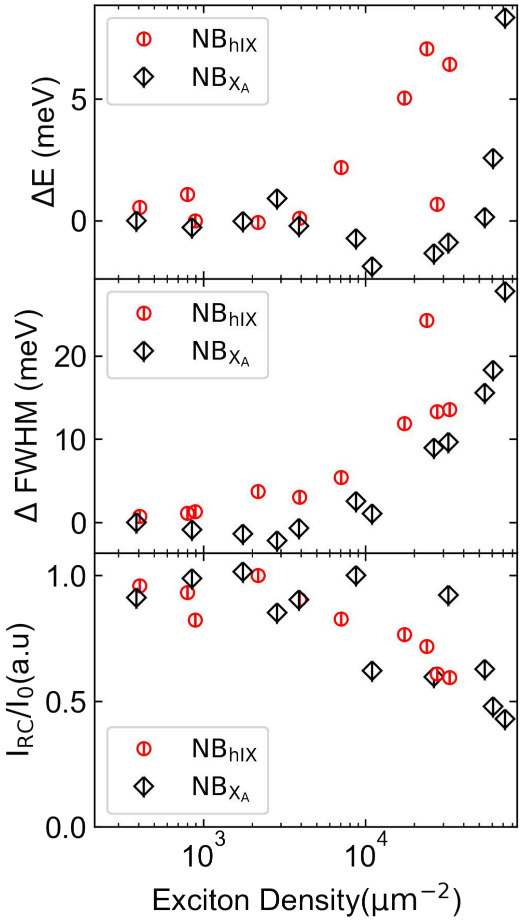

Supplementary Figure S11. From top to bottom: plots of the energy shift (ΔE), linewidth variation (ΔFWHM) and normalised integrated intensity (I_{RC}/I_0, whereI_0 is the maximum integrated intensity) as a function density for hIX (red marks) and X_A (black marks) peaks measured in RC. hIX is bleaching, broadening and blueshifting at slightly lower densities compared to X_A

SUPPLEMENTARY NOTE S8: MONOLAYER MoS_2 EXCITONS NONLINEAR BEHAVIOUR

The density dependent nonlinearity was studied for an encapsulated MoS_2 monolayer on an identical DBR substrate outside the cavity, in narrow band (NB) illumination regime (≈20 nm bandwidth). The results are then compared against the bilayer excitons excited with NB illumination (Supplementary Figure **S12**). A less significant bleaching of excitons in the monolayer is clearly apparent, confirming the theoretical predictions of increased interactions in bilayer excitons. Extrapolation such data, we estimate that the monolayer to reach complete bleaching at up to an order of magnitude higher densities.

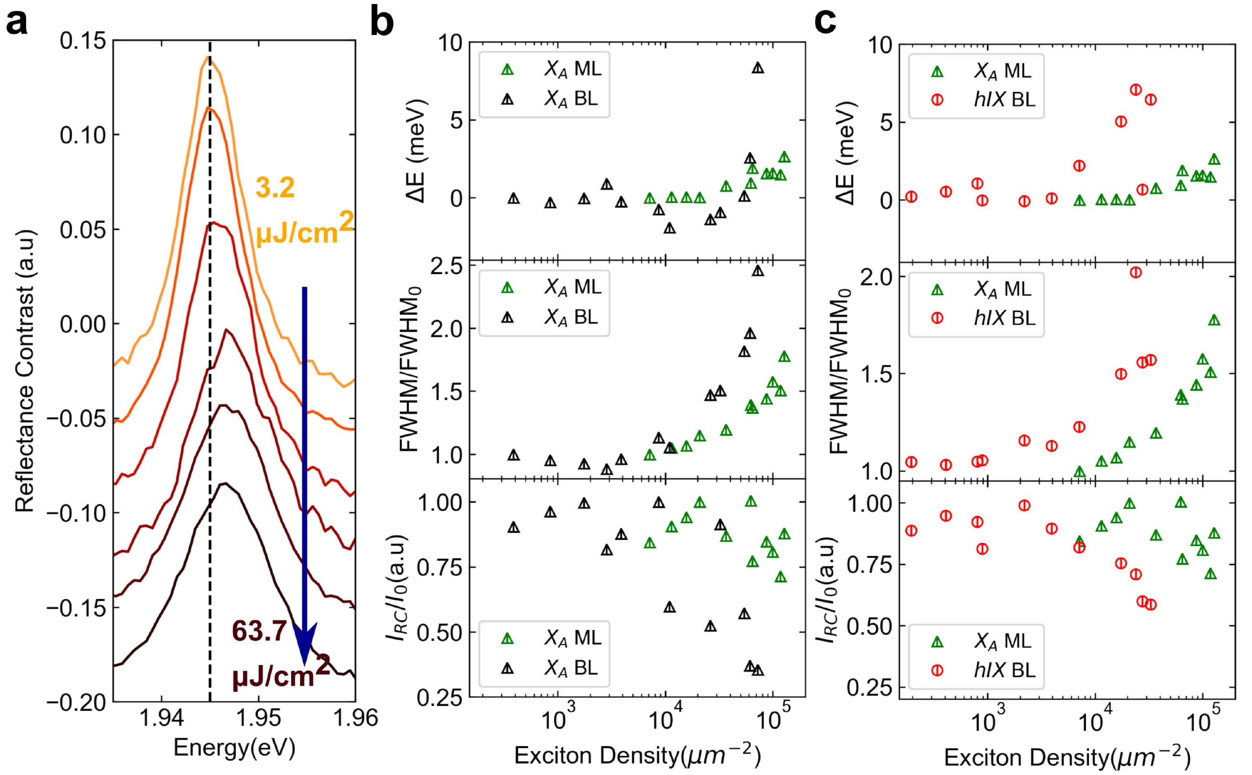

Supplementary Figure S12. **MoS_2 monolayer excitons nonlinear behaviour. a)** Waterfall of monolayer X_A RC spectra with the darker colours representing larger pump powers. Despite a blueshift is apparent,, the exciton bleaching is much less pronounced than for the bilayer excitons. **b, c)** Nonlinearity comparison of monolayer X_A to bilayer X_A **(b)** and hIX **(c)**. From top to bottom: plots of the energy shift (ΔE), normalised ($FWHM/FWHM_0$) and normalised integrated intensity (I_{RC}/I_0, whereI_0 is the maximum integrated intensity) as a function of exciton density, for the monolayer X_A (green), bilayer X_A(black) and hIX (red) under narrow band excitation.

SUPPLEMENTARY NOTE S9: THEORY OF NONLINEAR BROADENING AND PHASE SPACE FILLING

In our work we observed several nonlinear effects with contributions that depend on excitation conditions. In the BB regime, we already discussed the presence of two main species (flavours) of particles, namely direct or intralayer (X) and indirect or interlayer (IX) excitons, determining the features of the sample's optical response. This can be seen from the reflectance spectra in Fig. 3(a) and (b) of main text. We observe both the energy shifts (discussed above), and additional bleaching of the peaks. This hints that the presence of conservative nonlinear processes (energy shifts) is accompanied by dissipative nonlinear processes. Below, we discuss various contributions, which become enabled by the hole tunnelling. These include processes that involve Coulomb scattering, optical saturation due to phase space filling, and nonlinear change of non-radiative decay and dephasing processes.

We stress that in general all the aforementioned processes contribute to the spectral signal we observed. The peak shape is due to the competing contribution of radiative and non-radiative decay processes. The total broadening is $\Gamma = \Gamma_{\rm R} + \Gamma_{\rm NR}$ (where we can also add indices to label different modes). Both radiative $\Gamma_{\rm R}$ and non-radiative $\Gamma_{\rm NR}$ rates depend on the particle densities $n_{\rm D}$ and $n_{\rm I}$, for direct and interlayer excitons respectively. Here we simply refer to some generic density n, without specifying the particle flavour involved, as similar consideration apply to both. We relate the exciton radiative decay rate and the Rabi frequency Ω for polaritons, as they are both proportional to the particle oscillator strength $\Gamma_{\rm R}, \Omega \propto f_{\rm osc}$ [18]. More accurately, we know that $\Gamma_{\rm R} \propto f_{\rm osc}$, and $\Omega(n) \propto \sqrt{f_{\rm osc}}$. The oscillator strength of excitons depends on the number of particles due to Pauli exclusion [19], and can also be dependent on Coulomb interactions

and localisation [20]. In the lowest order, the collective oscillator strength linearly decreases with n, $\Omega(n) \approx \Omega_0(1 - \xi_{\rm sat}\alpha^2 n)$, where the nonlinear coefficient $\xi_{\rm sat}$ depends on the spatial size of excitons, $\propto \alpha^2$, and Ω_0 is the Rabi frequency in the low-occupation regime. This nonlinear interaction is dominant in the cavity-embedded samples showing the saturation of hIX-based dipolaritons (discussed in the end of this Note). Similarly, the radiative broadening can be written as $\Gamma_{\rm R}(n) \approx \Gamma_0(1 - g_{\rm sat}\alpha^2 n)$, and $g_{\rm sat} \sim \xi_{\rm sat}$. This effects the out-of-cavity samples and modifies the hybridization energies. We recall that the density-dependent splitting between $\mathrm{IX_B}$ and $\mathrm{X_B}$ modes can be written formally as $\Delta_{\rm hIX}(n) = \sqrt{4J_{\rm B}^2 + [E_{\rm X_B}(n) - E_{\rm IX_B}(n)]^2 - [\Gamma_{\rm R,X_B}(n) - \Gamma_{\rm R,IX_B}(n) + \Gamma_{\rm NR,X_B}(n) - \Gamma_{\rm NR,IX_B}(n)]^2}$. Due to the large difference of radiative lifetimes of intralayer and interlayer excitons, their difference $|\Gamma_{\rm R,X_B}(n) - \Gamma_{\rm R,IX_B}(n)|$ has a significant contribution in the low density regime, but the splitting between modes grows with n as we expand $\Gamma_{\rm R,X_B}(n) \approx \Gamma_{\rm R,X_B}(0)[1 - g_{\rm sat,X_B} n]$. This corresponds to the redshift of the UPB facilitated by the phase space filling and enabled by the hole tunnelling.

Another relevant process corresponds to the non-radiative losses, which plays a major role in bleaching. As there are many decay and dephasing channels, the full treatment of possible processes if formidable. Here, we address as the key effect the decay process due to Coulomb scattering [21]. As a result, the Coulomb scattering induced decay is proportional to the Coulomb scattering matrix

$$\Gamma_{\rm NR} \propto \sum_{\mathbf{q}\neq 0} |V^{\rm dir}(\mathbf{q}) - V^{\rm exch}(\mathbf{q})|^2 \delta(E(\mathbf{q}) + E(-\mathbf{q}) - 2E(\mathbf{Q} \sim 0)), \qquad \text{(S7)}$$

where E is the energy of involved particles and $V^{\rm dir,exch}(\mathbf{q})$ are direct and exchange particle scattering amplitudes, as discussed in the next Supplementary Note S10. Note that in the main text for brevity we refer to the combined effect of differ-

ent Coulomb-based processes using the combined interaction constant V_{Coul}. The discussed decay can be characterised by a spectral peak of the shape $\mathcal{L}(E)$ [22]

$$\mathcal{L}(E) = \frac{1}{\pi} \frac{\Gamma_{\mathrm{R}}(n)^2}{(E - E_0)^2 + [\Gamma_{\mathrm{R}}(n) + \Gamma_{\mathrm{NR}}(n)]^2}, \tag{S8}$$

where energy E is scanned through the exciton peak positioned at E_0. Analysing the experimental data with minimal square method, we estimate the non-radiative decay rate to be of the order of ~ 1 meV for direct excitons and ~ 10 meV for indirect excitons. The ratio between the two agrees well with the estimates for the interaction constants (see the discussion below). The corresponding plot is shown in Fig. **S13**, where coefficients ξ_{sat} and g_{sat} are of the order of ten and match experimental behaviour.

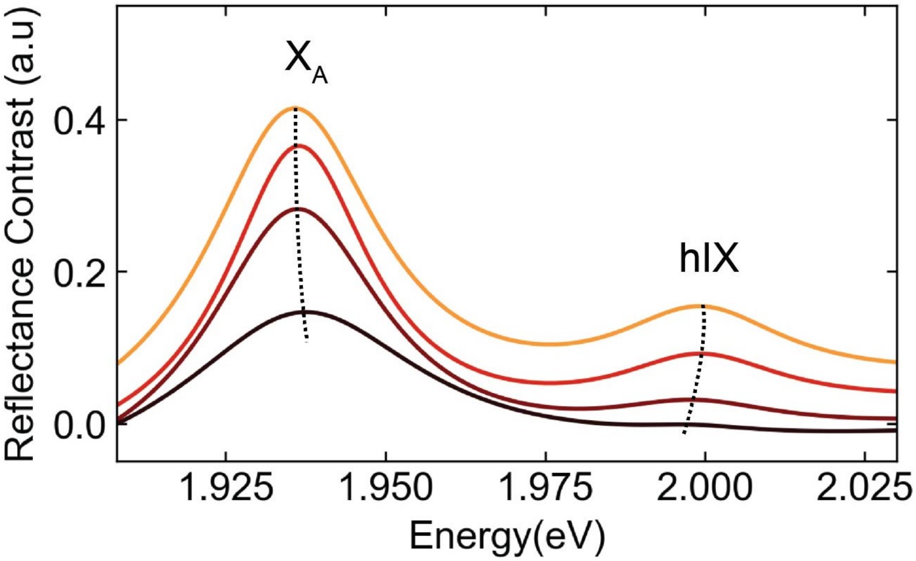

Supplementary Figure S13. **Nonlinear bleaching of the excitonic modes**. Reflectivity spectra for the broad band excitation case, modelled to reproduce qualitatively the observed experimental trends of the faster bleaching of hIX compared to X_A and the hIX redshift, the latter due to tunnelling-enabled phase-space filling.

Finally, we note that in the strong coupling case we need to combine both radiative and non-radiative bleaching. We find that the experimental Rabi splitting is well

described by the relation

$$\Omega(n) = \sqrt{\Omega_0^2(1 - \xi_{\rm sat}\alpha^2\, n)^2 - \Gamma_{\rm NR}^2(1 + \xi_{\rm NR}\alpha^2\, n)^2}, \tag{S9}$$

where the discussed experimental observation are well described by $\xi_{\rm NR} \sim 10$, and $\xi_{\rm sat} \sim 7$ describes the nonlinear saturation of the Rabi splitting. The origin of the saturation term comes from the interlayer exciton phase space filling, and is reminiscent to phase space filling effects discussed in Ref. [19].

SUPPLEMENTARY NOTE S10: THEORETICAL DISCUSSION ON INTERACTION CONSTANTS

In this Supplementary Note we consider microscopic mechanisms of exciton-exciton interactions. We discuss the properties of interaction constants as based on our analysis in Supplementary Note S1. The approach follows the exciton scattering formalism described in Refs. [23, 24]. With excitons being electron-hole bound states, the interaction among two separate excitons is mediated via Coulomb interaction of their elementary constituents. In particular, the full interaction potential $\Phi_{\text{tot}}(\mathbf{r}_\text{e}, \mathbf{r}_\text{h}, \mathbf{r'}_\text{e}, \mathbf{r'}_\text{e})$ is written as the sum of single-particle contributions, meaning $\Phi_{\text{tot}}(\mathbf{r}_\text{e}, \mathbf{r}_\text{h}, \mathbf{r}_{\text{e}'}, \mathbf{r}_{\text{h}'}) = \Phi(\mathbf{r}_\text{e}, \mathbf{r}_{\text{e}'}) + \Phi(\mathbf{r}_\text{e}, \mathbf{r}_{\text{h}'}) + \Phi(\mathbf{r}_\text{h}, \mathbf{r}_{\text{e}'}) + \Phi(\mathbf{r}_\text{h}, \mathbf{r}_{\text{h}'})$. Here, $\Phi(\mathbf{r}_\text{e}, \mathbf{r}_\text{h})$ is the Coulomb potential of two point-like particles and $\mathbf{r}_{\text{e,h}}$ are the position of electrons and holes, respectively. Due to Pauli exclusion principle, we introduce two separate contributions, namely *direct* $V^{\text{dir}}(\mathbf{Q}, \mathbf{q})$ and *exchange* $V^{\text{exch}}(\mathbf{Q}, \mathbf{q})$ potentials, both contributing to the total interaction. Here, $\mathbf{Q}$ and $\mathbf{q}$ are the exciton total momentum and the exchanged momentum, respectively. Formally, the two mentioned contributions read

$$V^{\text{dir}}(\mathbf{q}) = \int d^2r_\text{e}\, d^2r_\text{h}\, d^2r_{\text{e}'}\, d^2r_{\text{e}'} \phi^*(\mathbf{r}_\text{e}, \mathbf{r}_\text{h}) \phi^*(\mathbf{r}_{\text{e}'}, \mathbf{r}_{\text{h}'}) \Phi^{\text{tot}}(\mathbf{r}_\text{e}, \mathbf{r}_\text{h}, \mathbf{r}_{e'}, \mathbf{r}_{\text{h}'}) \phi(\mathbf{r}_\text{e}, \mathbf{r}_\text{h}) \phi(\mathbf{r}_{e'}, \mathbf{r}_{\text{h}'}), \tag{S10}$$

and

$$V^{\text{exch}}(\mathbf{q}) = \int d^2r_\text{e}\, d^2r_\text{h}\, d^2r_{\text{e}'}\, d^2r_{\text{e}'} \phi^*(\mathbf{r}_\text{e}, \mathbf{r}_\text{h}) \phi^*(\mathbf{r}_{\text{e}'}, \mathbf{r}_{\text{h}'}) \Phi^{\text{tot}}(\mathbf{r}_\text{e}, \mathbf{r}_\text{h}, \mathbf{r}_{e'}, \mathbf{r}_{\text{h}'}) \phi(\mathbf{r}_{e'}, \mathbf{r}_\text{h}) \phi(\mathbf{r}_\text{e}, \mathbf{r}_{\text{h}'}), \tag{S11}$$

where we make a physically justified assumption that the small total momentum for photoexcited excitons is small, $\mathbf{Q} \simeq \mathbf{0}$. For the specific case, we distinguish the two leading contributions of X-X and IX-IX interaction.

For intralayer excitons and the case of small exchanged momenta, the direct scat-

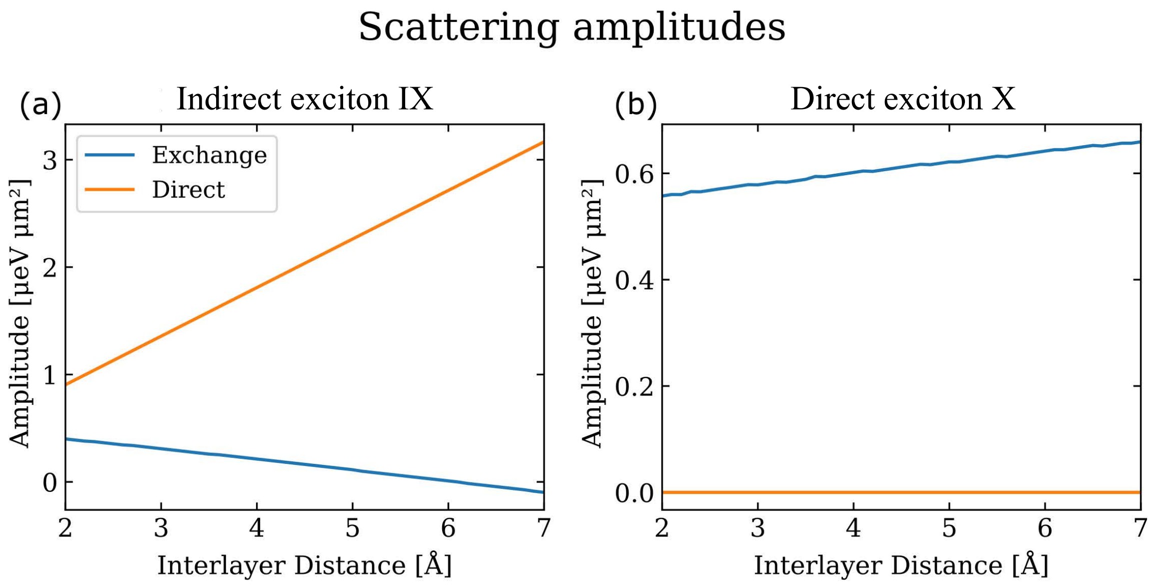

Supplementary Figure S14. **Scattering amplitudes for exciton-exciton Coulomb interaction.** **(a,b)** We show the change of scattering amplitudes with the interlayer distance. In **(a)**, note the behaviour of exchange scattering amplitude for indirect excitons, becoming negative past a threshold distance. Direct scattering amplitude grows linearly with distance. **(b)** We plot characteristic scattering amplitudes for direct excitons, where the direct process is zero at the negligible exchanged momentum (orange line), and the blue line corresponds to the exchange processes.

tering amplitude vanished, $V^{\rm dir}_{\rm X-X}(\mathbf{q}=\mathbf{0}) = 0$, and

$$\begin{aligned} V^{\rm exch}_{\rm X-X}(\mathbf{q}=\mathbf{0}) = &\left(\frac{2}{\pi}\right)^2 \frac{e^2}{4\pi\epsilon\epsilon_0}\alpha_D \int dx\,dy\,d\theta \frac{2\pi xy}{\delta(x,y,\theta)} \\ &\frac{1}{(1+r_0\delta(x,y,\theta)/\alpha_{\rm D})^2 - (\delta(x,y,\theta)r_0/\alpha_{\rm D})^2 e^{-2\delta(x,y,\theta)\frac{d}{\alpha_{\rm D}}}}\cdot \\ &\left(1+\frac{r_0}{\alpha_{\rm D}}\left(1-e^{-2\delta(x,y,\theta)\frac{d}{\alpha_{\rm D}}}\right)\right)\cdot\left(\frac{(-1)}{(1+x^2)^3}\frac{1}{(1+y^2)^3}\right. \\ &\left.+\frac{1}{(1+x^2)^3}\frac{e^{-\delta(x,y,\theta)\frac{d}{\alpha_{\rm D}}}}{(1+y^2)^{3/2}(1+x^2)^{3/2}}\right), \end{aligned} \tag{S12}$$

where $\delta(x,y,\theta) = \sqrt{x^2+y^2-2xy\cos\theta}$. With indirect excitons, the direct scattering amplitude in homobilayers recovers the parallel plate capacitor formula $V^{\rm dir}_{\rm I-I}(\mathbf{q} = \mathbf{0}) = e^2/(\epsilon\epsilon_0 A)d$, where A is the sample area. Finally, the indirect exciton exchange

potential has to be evaluated as

$$V_{\mathrm{I-I}}^{\mathrm{exch}}(\mathbf{q}=\mathbf{0}) = \left(\frac{2}{\pi}\right)^2 \frac{e^2}{4\pi\epsilon\epsilon_0}\alpha_D \int dx\, dy\, d\theta \frac{2\pi xy}{\delta(x,y,\theta)} \frac{1}{(1+r_0\delta(x,y,\theta)/\alpha_{\mathrm{I}})^2 - (\delta(x,y,\theta)r_0/\alpha_{\mathrm{I}})^2 e^{-2\delta(x,y,\theta)\frac{d}{\alpha_{\mathrm{I}}}}} \cdot \left[\frac{(-1)}{(1+x^2)^3}\frac{1}{(1+y^2)^3}\left(1+\frac{r_0}{\alpha_{\mathrm{I}}}\left(1-e^{-2\delta(x,y,\theta)\frac{d}{\alpha_{\mathrm{I}}}}\right)\right) + \frac{1}{(1+x^2)^3}\frac{e^{-\delta(x,y,\theta)\frac{d}{\alpha_{\mathrm{I}}}}}{(1+y^2)^{3/2}(1+x^2)^{3/2}}\right]. \tag{S13}$$

Figs. **S14**(a) and (b) show the dependence of scattering amplitudes on the interlayer distance. We note the behaviour of exchange scattering amplitude for indirect excitons, becoming negative past threshold distance [see Fig. **S14**(a)]. Characteristically, for intralayer (i.e. direct) excitons we have a zero direct scattering amplitude, with non-zero contributions only due to the particle exchange process. With the estimated parameters, we find interlayer exciton-exciton interaction to have a scattering constant of $V_{\mathrm{I-I}}(\mathbf{q}=\mathbf{0}) \simeq 2.5$ μeV μm^2, setting the scale for the Coulomb-based interactions.

Next, let us consider a complete symmetric geometry of the system. With probing both layers, we excite direct excitons in both layers of the homobilayer, as well as indirect excitons with same and opposite dipole orientations. In the absence of asymmetry densities of photocreated excitons are equal. However, the effective interaction constants are different. Namely, excitons with opposite dipole orientation have no exchange contributions, and opposite direct interaction term. This is accounted in the energy shifts for the modes. Second, as implied by Eq. (S7), all interaction contributions (positive and negative) lead to peak broadening, and play relevant role for bleaching (see Supplementary Note S11).

Finally, let us comment on the intralayer nonlinear processes. For $\mathrm{X_A}$ excitons the

nonlinearity coefficient scales as $g_{\mathrm{X-X}} \sim a_{\mathrm{B}}$ [19], being the relevant scale for Coulomb exchange processes that dominate low momentum $\mathrm{X_A}$-$\mathrm{X_A}$ scattering, where direct processes are negligible. On the other hand, for interlayer excitons dipolar interaction leads to the significant direct IX-IX scattering contribution. The scaling of IX-IX nonlinearity that linearly grows with the out-of-plane dipole moment, $g_{\mathrm{IX-IX}} \sim d$, where d is the electron-hole separation distance. This is in line with dipolar exciton behaviour [23, 25]. At the same time, due to the small absolute values of e-h separation in homobilayers, being estimated as $d = 0.55$ nm and smaller than $a_{\mathrm{B}} \sim 2$ nm, we note that exchange processes remain important for the hIX mode, and more so as a part of cross-mode scattering.

SUPPLEMENTARY NOTE S11: THEORY FOR ENERGY SHIFTS

The out-of-cavity measurements (see Fig. 3 main text) show different behaviour according to the operating regime, namely narrow bandwidth (NB) and broad bandwidth (BB). In NB regime, we predominantly excite only one particle flavour, depending on targeting spectral regions corresponding to either intralayer (X_A) or hybridised interlayer (hIX) excitons. In this case we observe a blueshift of peaks as expected from exchange interactions [23, 26, 27]. On the other hand, in the BB regime, where we excite both flavours ($X_{A,B}$ and thus hIX), we observe hIX peak' s redshift. Here, we describe nonlinear contributions from the phase space filling effects and Coulomb interactions, based on the contributions to the total energy of the system. Specifically, we compute the system energy as the expectation value of the system Hamiltonian $\hat{\mathcal{H}}$ on a many-body state, keeping track of the non-bosonicity and composite structure of excitons. For the current analysis, we concentrate on one exciton configuration, and note that the result may be generalised to other cases as well superpositions of states.

We consider electrons and holes distributed in a homobilayer as shown in Fig. 1(d) [main text]. We denote $N_{\mathrm{I(D)}}$ as the number of indirect (direct) excitons in the sample, and $n_{\mathrm{I(D)}}$ as the particle density. The spin indices are omitted for brevity. The Hamiltonian of the system can be written as

$$\hat{\mathcal{H}} = \hat{\mathcal{H}}_{\mathrm{T}} + \hat{\mathcal{H}}_{\mathrm{sm}} + \hat{\mathcal{H}}_{\mathrm{df}}, \tag{S14}$$

where $\hat{\mathcal{H}}_{\mathrm{T}}$ is the kinetic term, $\hat{\mathcal{H}}_{\mathrm{sm}}$ and $\hat{\mathcal{H}}_{\mathrm{df}}$ are the Coulomb interactions. With $\hat{\mathcal{H}}_{\mathrm{sm}}$ we refer to interacting particles belonging to same band, and $\hat{\mathcal{H}}_{\mathrm{df}}$ corresponds

to different dispersion bands. Explicitly, the kinetic energy reads

$$\hat{\mathcal{H}}_{\mathrm{T}} = \sum_{\mathbf{k}} \left[\varepsilon_c^t(\mathbf{k})\hat{a}^\dagger_{\mathbf{k}}\hat{a}_{\mathbf{k}} + \varepsilon_v^t(\mathbf{k})b^\dagger_{\mathbf{k}}b_{\mathbf{k}} + \varepsilon_c^b(\mathbf{k})c^\dagger_{\mathbf{k}}c_{\mathbf{k}}\right], \tag{S15}$$

where $\hat{a}^\dagger_{\mathbf{k}}$ and $\hat{b}^\dagger_{\mathbf{k}}$ are creation operators for conduction and valence bands of the top layer, respectively. $\hat{c}^\dagger_{\mathbf{k}}$ is an electron annihilation operator for the conduction band of the bottom layer. Each operator is labelled with a crystal momentum $\mathbf{k}$. $\varepsilon_{\mathrm{c(v)}}^{\mathrm{t(b)}}(\mathbf{k})$ are dispersions for conduction (valence) bands in the top (bottom) layer. The interactions are mediated through the Keldysh-Rytova potential. The corresponding interaction Hamiltonian reads

$$\hat{\mathcal{H}}_{\mathrm{sm}} = \frac{1}{2}\sum_{\mathbf{k},\mathbf{k}',\mathbf{q}} \left[V_{\mathrm{KR}}^{\mathrm{intra}}(\mathbf{q})(a^\dagger_{\mathbf{k}-\mathbf{q}}\hat{a}^\dagger_{\mathbf{k}'+\mathbf{q}}\hat{a}_{\mathbf{k}'}\hat{a}_{\mathbf{k}} + \hat{b}^\dagger_{\mathbf{k}-\mathbf{q}}\hat{b}^\dagger_{\mathbf{k}'+\mathbf{q}}\hat{b}_{\mathbf{k}'}\hat{b}_{\mathbf{k}} + c^\dagger_{\mathbf{k}-\mathbf{q}}\hat{c}^\dagger_{\mathbf{k}'+\mathbf{q}}\hat{c}_{\mathbf{k}'}\hat{c}_{\mathbf{k}})\right], \tag{S16}$$

$$\hat{\mathcal{H}}_{\mathrm{df}} = \sum_{\mathbf{k},\mathbf{k}',\mathbf{q}} \left[V_{\mathrm{KR}}^{\mathrm{inter}}(\mathbf{q})(\hat{a}^\dagger_{\mathbf{k}-\mathbf{q}}\hat{c}^\dagger_{\mathbf{k}'+\mathbf{q}}\hat{c}_{\mathbf{k}'}\hat{a}_{\mathbf{k}} + \hat{b}^\dagger_{\mathbf{k}-\mathbf{q}}\hat{c}^\dagger_{\mathbf{k}'+\mathbf{q}}\hat{c}_{\mathbf{k}'}\hat{b}_{\mathbf{k}}) + V_{\mathrm{KR}}^{\mathrm{intra}}\hat{a}^\dagger_{\mathbf{k}-\mathbf{q}}\hat{b}^\dagger_{\mathbf{k}'+\mathbf{q}}\hat{b}_{\mathbf{k}'}\hat{a}_{\mathbf{k}}\right]. \tag{S17}$$

We define the exciton creation operators in the form

$$\hat{D}^\dagger_\mu(\mathbf{Q}) = \sum_{\mathbf{k}} \phi^\mu_{\mathrm{D}}(\mathbf{k})\hat{a}^\dagger_{\mathbf{k}+\gamma_{\mathrm{e}}\mathbf{Q}}\hat{b}_{\mathbf{k}-\gamma_{\mathrm{h}}\mathbf{Q}}, \tag{S18}$$

$$\hat{I}^\dagger_\nu(\mathbf{P}) = \sum_{\mathbf{k}} \phi^\nu_{\mathrm{I}}(\mathbf{k})\hat{a}^\dagger_{\mathbf{k}+\gamma_{\mathrm{e}}\mathbf{P}}\hat{c}_{\mathbf{k}-\gamma_{\mathrm{h}}\mathbf{P}}, \tag{S19}$$

with $\hat{D}^\dagger_\mu(\mathbf{Q})$ [$\hat{I}^\dagger_\nu(\mathbf{P})$] and $\phi^\mu_{\mathrm{D}}(\mathbf{k})$ [$\phi^\nu_{\mathrm{I}}(\mathbf{k})$] being the direct [indirect] exciton creation operator and wave function, respectfully. $\mathbf{Q}, \mathbf{P}$ are crystal momenta, and μ, ν are state indices. When we omit the μ and ν indices, and the total momentum, we refer the ground state at crystal momentum $\mathcal{Q} = 0$. To take into account for particle non-bosonicity and consequent nonlinear behaviour, we consider the expectation value of a system over a multi-particle state created by exciting the vacuum state $|\Omega_0\rangle$. It includes N_{D} direct excitons and N_{I} indirect excitons. The expectation $\langle\Omega_0|\hat{D}^{N_{\mathrm{D}}}\hat{I}^{N_{\mathrm{I}}}\hat{\mathcal{H}}\hat{D}^{\dagger N_{\mathrm{D}}}\hat{I}^{\dagger N_{\mathrm{I}}}|\Omega_0\rangle$ then denotes the total energy of the many-body system.

By following a procedure for accounting non-bosonic correction at increasing order [28], we first commute the Hamiltonian with the product of exciton operators $\hat{D}^{\dagger N_{\mathrm{D}}}$, leading to

$$[\mathcal{H}, \hat{D}^{\dagger N_{\mathrm{D}}}] = N_{\mathrm{D}} E_{\mathrm{D}} \hat{D}^{\dagger N_{\mathrm{D}}-1} + N_{\mathrm{D}} \hat{D}^{\dagger N_{\mathrm{D}}-1} \hat{V}_{\mathrm{D}} + \frac{N_{\mathrm{D}}(N_{\mathrm{D}}-1)}{2} \hat{D}^{\dagger N_{\mathrm{D}}-2} \sum_{\mu,\nu,\mathrm{q}} V^{\mu,\nu}_{\mathrm{D-D}}(\mathbf{q}) \hat{D}^{\dagger}_{\nu}(\mathbf{q}) \hat{D}^{\dagger}_{\mu}(-\mathbf{q}), \tag{S20}$$

where $V^{\mu,\nu}_{\mathrm{D-D}}(\mathbf{q})$ is the scattering matrix element of two direct excitons exchanging a momentum of $\mathbf{q}$. $\hat{V}_{\mathrm{D}}$ is the scattering potential [28], which arises from the commutator $[\mathcal{H}, \hat{D}^{\dagger}]$ and is a signature of the phase space filling. Similarly, for indirect excitons we get

$$[\mathcal{H}, \hat{I}^{\dagger N_{\mathrm{I}}}] = N_{\mathrm{I}} E_{\mathrm{I}} \hat{I}^{\dagger \mathrm{N_I}-1} + N_{\mathrm{I}} \hat{I}^{\dagger N_{\mathrm{I}}-1} \hat{V}_{\mathrm{I}} + \frac{N_{\mathrm{I}}(N_{\mathrm{I}}-1)}{2} \hat{I}^{\dagger \mathrm{N_I}-2} \sum_{\mu,\nu,\mathrm{q}} V^{\mu,\nu}_{\mathrm{I-I}}(\mathbf{q}) \hat{I}^{\dagger}_{\nu}(\mathbf{q}) \hat{I}^{\dagger}_{\mu}(-\mathbf{q}), \tag{S21}$$

with the notation being similar to Eq. (S20). We note the property of the scattering potential such that $\hat{V}_{\mathrm{D,I}}|\Omega_0\rangle = 0$. With the given commutators, we can rewrite the total energy as

$$\begin{aligned} &\langle \Omega_0 | \hat{D}^{N_{\mathrm{D}}} \hat{I}^{N_{\mathrm{I}}} \hat{\mathcal{H}} \hat{D}^{\dagger N_{\mathrm{D}}} \hat{I}^{\dagger N_{\mathrm{I}}} | \Omega_0 \rangle = \\ &= N_{\mathrm{I}} E_{\mathrm{I}} \langle \Omega_0 | \hat{D}^{N_{\mathrm{D}}} \hat{I}^{N_{\mathrm{I}}} \hat{D}^{\dagger N_{\mathrm{D}}} \hat{I}^{\dagger N_{\mathrm{I}}} | \Omega_0 \rangle + N_{\mathrm{D}} E_{\mathrm{D}} \langle \Omega_0 | \hat{I}^{N_{\mathrm{I}}} \hat{D}^{N_{\mathrm{D}}} \hat{D}^{\dagger N_{\mathrm{D}}} \hat{I}^{\dagger N_{\mathrm{I}}} | \Omega_0 \rangle \\ &+ \frac{N_{\mathrm{D}}(N_{\mathrm{D}}-1)}{2} \sum_{\mu,\nu,\mathrm{q}} V^{\mu,\nu}_{\mathrm{D-D}}(\mathbf{q}) \langle \Omega_0 | \hat{D}^{N_{\mathrm{D}}} \hat{I}^{N_{\mathrm{I}}} \hat{D}^{\dagger N_{\mathrm{D}}-2} \hat{D}^{\dagger}_{\nu}(\mathbf{q}) \hat{D}^{\dagger}_{\mu}(-\mathbf{q}) \hat{I}^{\dagger N_{\mathrm{I}}} | \Omega_0 \rangle \\ &+ \frac{N_{\mathrm{I}}(N_{\mathrm{I}}-1)}{2} \sum_{\mu,\nu,\mathrm{q}} V^{\mu,\nu}_{\mathrm{I-I}}(\mathbf{q}) \langle \Omega_0 | \hat{D}^{N_{\mathrm{D}}} \hat{I}^{N_{\mathrm{I}}} \hat{D}^{\dagger N_{\mathrm{D}}} \hat{I}^{\dagger N_{\mathrm{I}}-2} \hat{I}^{\dagger}_{\nu}(\mathbf{q}) \hat{I}^{\dagger}_{\mu}(-\mathbf{q}) | \Omega_0 \rangle \\ &+ N_{\mathrm{D}} N_{\mathrm{I}} \sum_{\mu,\nu,\mathbf{q}} V^{\mu,\nu}_{\mathrm{D-I}}(\mathbf{q}) \langle \Omega_0 | \hat{D}^{N_{\mathrm{D}}} \hat{I}^{N_{\mathrm{I}}} \hat{D}^{\dagger N_{\mathrm{D}}-1} \hat{D}^{\dagger}_{\mu}(\mathbf{q}) \hat{I}^{\dagger}_{\nu}(-\mathbf{q}) \hat{I}^{\dagger N_{\mathrm{I}}-1} | \Omega_0 \rangle. \end{aligned} \tag{S22}$$

Eq. (S22) describes various energy contributions (linear and nonlinear) that are

present in the system. We note that each term is proportional to the expectation value $\langle\Omega_0|\hat{D}^{N_\mathrm{D}}\hat{I}^{N_\mathrm{I}}\hat{D}^{\dagger N_\mathrm{D}}\hat{I}^{\dagger N_\mathrm{I}}|\Omega_0\rangle$, which deviates from 1 due to the non-bosonicity of composite excitons. This reveals three possible creation potentials $\hat{V}^{\mu,\nu}_{\mathrm{D-D}}, \hat{V}^{\mu,\nu}_{\mathrm{I-I}}$, and $\hat{V}^{\mu,\nu}_{\mathrm{D-I}}$, which noticeably generate cross-flavour interaction. Coulomb terms in Eq. (S22) are the non-linear energy shift of excitons, and these terms are responsible for peaks shift. Referring to Eq. (S22), line 2 is the energy of system particles growing linearly with the occupation, as expected in systems with no interaction. Lines 3 and 4 are the contributions of DX-DX (3) and hIX-hIX (4) interaction, and the last term in line 5 is the cross interaction of direct and indirect excitons. In the full symmetric picture (see Fig. **S1**), the latter has both contributions of hole exchange in valence band, and partial dipole moment of DX being oriented in opposite direction. The effect is proportional to the out of plane dipole moment of DX as estimated in Supplementary Note 1. The effect of intra-flavour terms has already been discussed in Ref. [19], and for the intralayer modes leads to energy blueshifts. Finally, the discussed contributions are summed up to the already discussed blueshift in Supplementary Note S9 coming from renormalization of decay rates. We refer to the attraction due to holes being shared between DX and IX mode as tunnelling-enabled phase space filling effect.

Next, we proceed to evaluating intergrals for the described matrix elements. We consider the relevant limit of small exchanged momenta $\mathbf{q} \simeq 0$, as it gives the dominant contribution observed in experiments, and we take particles being in the ground state as a main occupation at low temperatures. With this, we rewrite the mutual energy shift $\Delta E_\mathrm{I} = \sum_{\mu,\nu,\mathbf{q}} V^{\mu,\nu}_{\mathrm{D-I}}(\mathbf{q})\langle\Omega_0|\hat{D}^{N_\mathrm{D}}\hat{I}^{N_\mathrm{I}}\hat{D}^{\dagger N_\mathrm{D}-1}\hat{D}^{\dagger}_{\mu}(\mathbf{q})\hat{I}^{\dagger}_{\nu}(-\mathbf{q})\hat{I}^{\dagger N_\mathrm{I}-1}|\Omega_0\rangle$ as

$$\Delta E_\mathrm{I} = \xi\frac{e^2}{4\pi\epsilon\epsilon_0 A}\mathcal{I}\alpha, \tag{S23}$$

with A being the sample area, and parameter ξ of the order of unity, $\xi \sim 1$, is tuned

to match with experimental data setting an effective area. Here, $\alpha = (\alpha_{\mathrm{D}}^{-1} + \alpha_{\mathrm{I}}^{-1})^{-1}$ is the reduced particle Bohr radius. The dimensionless exchange integral $\mathcal{I}$ has the form

$$\begin{aligned}\mathcal{I} = \int d^2x d^2y \tilde{V}_{\mathrm{KR}}^{\mathrm{intra}}(|\mathbf{x}-\mathbf{y}|) &\frac{1}{(1+\alpha_1^2x^2)^{3/2}}\frac{1}{(1+\alpha_2^2y^2)^3}\cdot \\ &\cdot\left(\frac{1}{(1+\alpha_1^2x^2)^{3/2}} - \frac{1}{(1+\alpha_2^2y^2)^{3/2}}\right) \\ &+ \tilde{V}_{\mathrm{KR}}^{\mathrm{inter}}(|\mathbf{x}-\mathbf{y}|)\frac{1}{(1+\alpha_2^2y^2)^{3/2}}\frac{1}{(1+\alpha_1^2y^2)^{3/2}}\frac{1}{(1+\alpha_2^2x^2)^{3/2}}\cdot \\ &\cdot\left(\frac{1}{(1+\alpha_1^2x^2)^{3/2}} - \frac{1}{(1+\alpha_1^2y^2)^{3/2}}\right),\end{aligned} \tag{S24}$$

where $\tilde{V}_{\mathrm{KR}}^{\mathrm{inter}}(|\mathbf{x}-\mathbf{y}|)$ is Keldysh-Rytova potential in dimensionless form, α_1 and α_2 are respectfully equal to $\alpha_{\mathrm{D}}/\alpha$ and $\alpha_{\mathrm{I}}/\alpha$. By estimating the exciton density as proportional to oscillator strength, we find $n_{\mathrm{I}} \simeq n_{\mathrm{D}}/4$. Numerically, we estimate the shift with $\epsilon = 4$, $\alpha \simeq 6.7$ Å. The resulting shift can be estimated as $\Delta E_{\mathrm{I}} = -0.24\xi$ μeV μm$^2\sqrt{n_{\mathrm{D}}n_{\mathrm{I}}}$.

Finally, we consider a bilayer system accounting for some potential asymmetry, which typically arises due to imperfections [Fig. **S1**(a)]. For the symmetric samples, excitons that interact with parallel and anti-parallel dipole moment orientations induce nonlinear shifts with the same magnitude and opposite signs. This negates the two contributions, leading to vanishing real energy shifts. Here, we discuss the specific case of NB, and generalize as the same argument applies in BB. As already discussed at the end of Sec. S9, we consider two effects of peak broadening and shift. The extra contributions enhance peak broadening, because the Coulomb driven decay rate is proportional to the absolute value of interaction. However, the effect over the peak positions is different. In the current experimental setup, we assume the sample presents some symmetry breaking field (i.e. electrostatic field due to wetting layers), and the total effect unbalances the number of particles in

the sample, so we have $n + \delta n$ particles oriented in one direction and $n - \delta n$ in the opposite one. In this case, the total energy shift ΔE can be written as

$$\Delta E = V_{\text{dip}}\left(\frac{(n+\delta n)^2 + (n-\delta n)^2}{2} - (n-\delta n)(n+\delta n)\right) = 2V_{\text{dip}}\delta n^2, \tag{S25}$$

where V_{dip} is the hIX-hIX matrix element of interaction. As shown, asymmetry in the system leads to renormalized contribution being proportional to the difference in particle population. With the effects being qualitatively the same, we conclude that the symmetry of the system is broken leading to the observation of same qualitative phenomena.

SUPPLEMENTARY NOTE S12: RELATIVE POPULATION OF EXCITONS

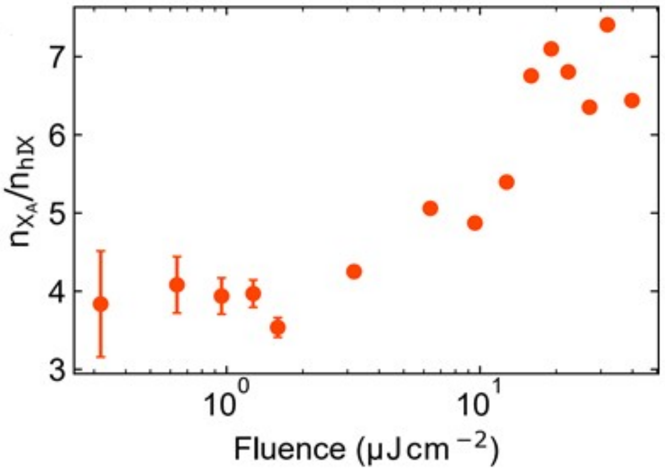

Supplementary Figure S15. Ratio of intralayer to interlayer exciton density measured (n_{X_A}/n_{hIX}) as a function of pump fluence.

As discussed in the main text and the previous supplementary sections, the tunneling-enabled nonlinear effects on one exciton (X_A, hIX) depends on the population of the other exciton n_{hIX}, n_{X_A}. Fig **S15** shows the intralayer to interlayer exciton density at a given fluence exciting the sample with BB illumination, for the range of fluences used. As the fluence increases, the relative population of intralayer excitons grows significantly, increasing the imbalance of X_A population, explaining why the BB illumination results in different effects for X_A and hIX, becoming very similar to the case of NB illumination for the former, while being strongly dependent on the opposite species for the latter.

SUPPLEMENTARY NOTE S13: FLUENCE DEPENDENCE ON GLOVEBOX SAMPLE ON SI/SIO$_2$

Here we show the fluence dependence data taken on a high-quality sample on Si/SiO$_2$, fabricated in an inert environment. The results in Fig **S16**, show qualitatively the same behaviour as the one shown in the main text, with the effect of BB illumination (tunneling-enabled nonlinearity) still pronounced.

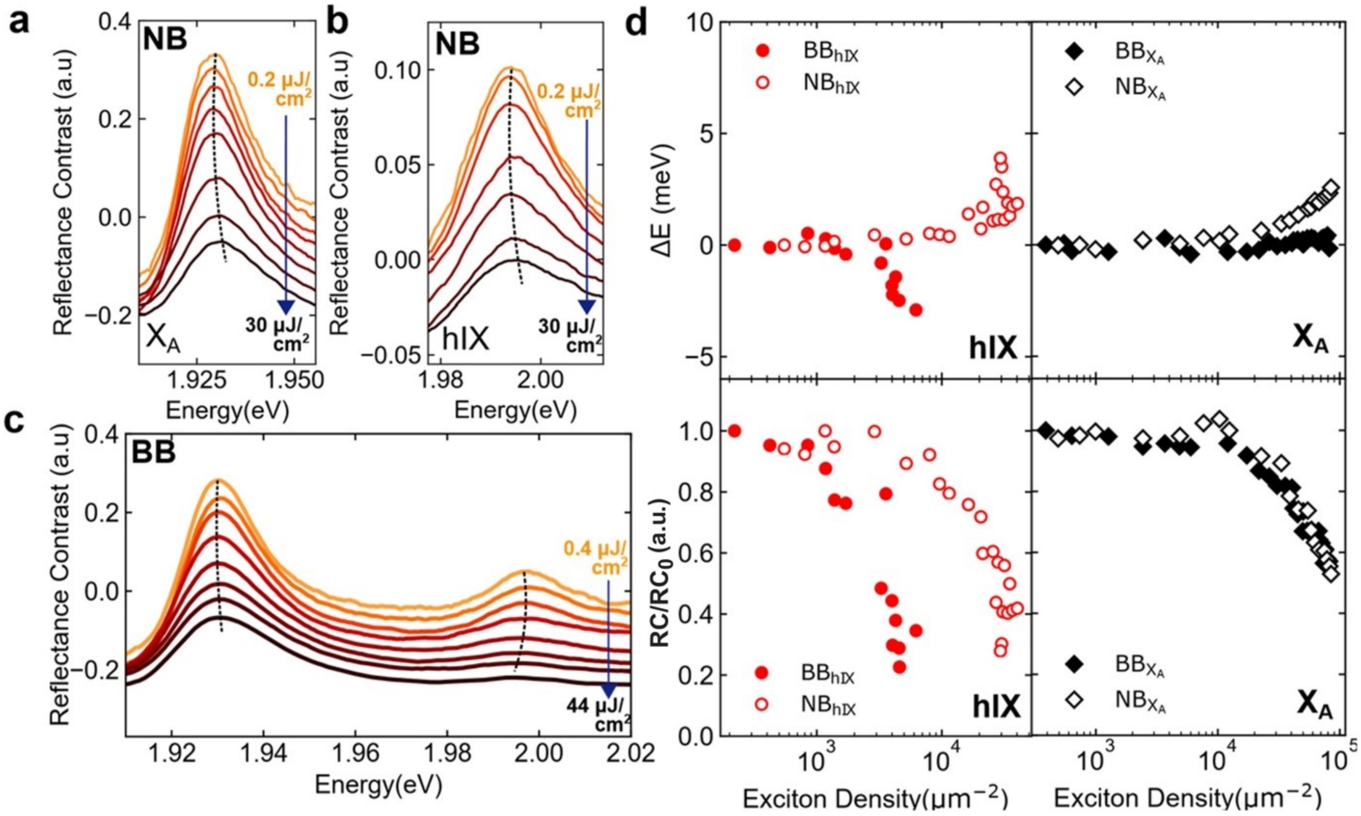

Supplementary Figure S16. Exciton nonlinearity in high quality MoS$_2$ bilayer on Si/SiO$_2$. **a,b**, RC spectra measured with the NB(FWHM=28nm) excitation for the X_A (**a**) and hIX (**b**), and with the BB (FWHM=50nm) excitation (c) at different fluences. The dashed curves are guide for the eye. d, e, The energy shift ΔE (top) and normalized integrated intensity (bottom) as a function of the exciton density for the hIX (black) and X_A (black). Solid (open) symbols show the results for the BB (NB) excitation. For the normalized RC we divide the spectrally integrated RC at each laser fluence by its maximum value.

We note that, as shown in Fig **S17**, owing to the inert environment and a thicker hBN on a cleaner and flatter substrate, inhomogeneous broadening is reduced. We ,therefore, reproduce our results in two samples with notably different inhomogeneous broadening, which indicates that qualitatively the inhomogeneous broadening

does not play a significant role.

**Exciton**	**Energy(eV)**	**Linewidth(meV)**	**Amplitude(a.u.)**
$\mathbf{X}_{\mathrm{A}}$	1.926	14	4.8
**hIX**	1.993	17	1.7
$\mathbf{hX}_{\mathrm{B}}$	2.107	51	6.6

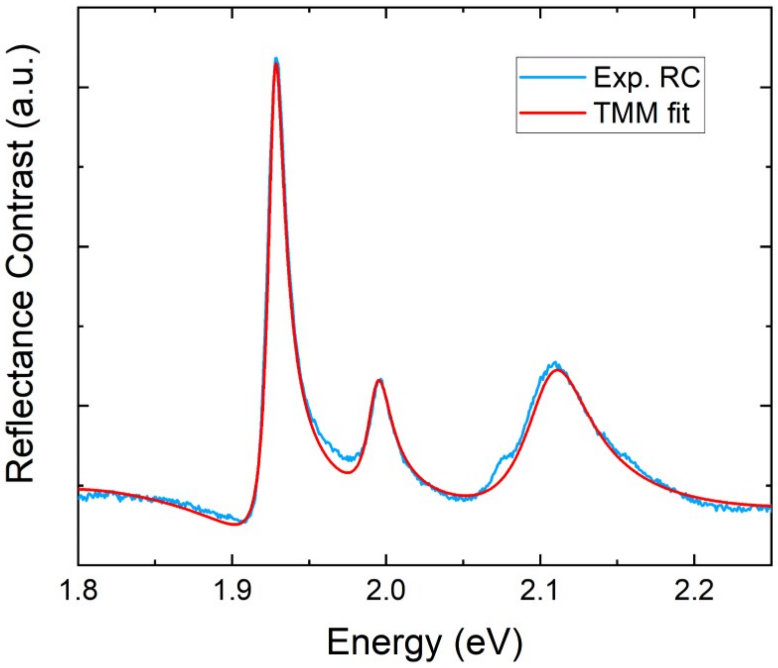

Supplementary Figure S17. Fit of the experimental RC measured on the MoS_2 bilayer on Si/SiO_2, performed using the Transfer Matrix Method (TMM). The complex refractive index of the BL used in the TMM simulation was modelled with three Lorentzians, whose amplitudes, peak energies and FWHM were kept as free parameters for the fit.

SUPPLEMENTARY NOTE S14: NONLINEARITY COMPARISON TO MONOLAYER POLARITONS AND OTHER SYSTEMS

Here we compare the enhancement of nonlinear coefficient found in our work with other systems. The exact values of nonlinearity factors depend heavily on the method for density estimation and the cavity system used in each study. It is, therefore, more instructive to compare in each study separately the enhancement or ratio of the nonlinearity factors measured in the proposed system with respect to those in monolayer neutral exciton-polaritons. Here we show the measurement of nonlinearity on neutral intralayer excitons of an encapsulated MoS_2 monolayer embedded in a microcavity of the same structure, which serves as a reference for comparison. In this case the nonlinear coefficient of monolayer polaritons was 0.05 μeVμm^2 (see Figure **S18**), which is more than one order of magnitude lower than for the bilayer polaritons.

Regarding the previous works on trion-polaritons [19], comparing the reported data at densities where strong coupling exists for both trions and neutral excitons (10^2-10^3 μm^{-2}), we can see that the non-linearity values are 37 μeVμm^2 for trion-polaritons compared to ≈ 1.5 μeVμm^2 for neutral exciton-polaritons, giving a ratio comparable to what we found for dipolaritons in BL MoS2. In the recent publication about moire' polaritons [16], a nonlinearity of ≈ 0.4 μeVμm^2 for moire polaritons was shown, compared to 0.04 μeVμm^2 for monolayer neutral polaritons, giving a maximum enhancement of an order of magnitude, in the same density range that we report. Moreover, in another recent work about 2s-polaritons [29] the authors mention *"The associated interaction strength of 2s exciton-polaritons is shown to be ~ 4.6 times larger than that of 1s exciton-polaritons"* comparing their reported nonlinearity of 2s (46.3±13.9 μeVμm^2) to the neutral exciton (10±4.2

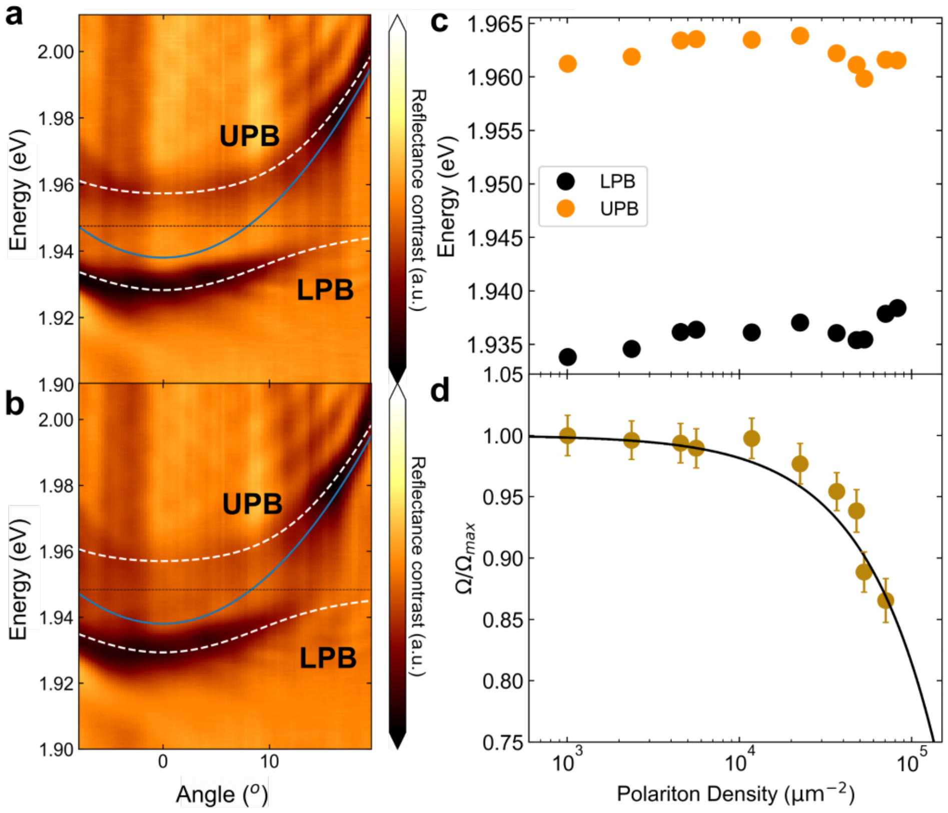

Supplementary Figure S18. Monolayer intralayer polariton dispersion at low(**a**) and high(**b**) fluence. (**b**) Measured LPB and UPB energies at resonance as a function of polariton density. Symbols show the Rabi splittings normalized by the Rabi splitting measured at the lowest power ($\Omega/\Omega_{\mathrm{max}}$). The line shows the fitting using our theoretical model.

μeVμm^2). We therefore conclude that our samples show improved nonlinearity or at least comparable to other 2D systems, featuring in addition a higher Rabi splitting. Indeed, comparing the extracted Rabi splitting (19 meV) with other nonlinear systems in planar Ag/DBR cavities in literature (7.7 meV for 2s-polaritons and 8.5-10 meV for moiré polaritons), MoS_2 bilayers combine both high nonlinearity and large coupling strength.

Regarding other more conventional systems, single or few GaAs quantum well polaritons [30, 31] show nonlinearity of about 3 μeVμm^2 , but with much lower Rabi splittings and much lower temperature stability. Noting the differences in the methods used to calculate the nonlinearity reported in the literature, we see that hIX are less than 10 times lower than conventional GaAs exciton with the possibility to

surpass them with further advances, showing exceptional non-linearity for strongly bound TMD excitons.

* charalambos.louca@polimi.it; These two authors contributed equally
† armando.genco@polimi.it; These two authors contributed equally
‡ a.tartakovskii@sheffield.ac.uk

[1] E. Cappelluti, R. Roldán, J. A. Silva-Guillén, P. Ordejón, and F. Guinea, Tight-binding model and direct-gap/indirect-gap transition in single-layer and multilayer mos_2, Phys. Rev. B **88**, 075409 (2013).

[2] P. Cudazzo, I. V. Tokatly, and A. Rubio, Dielectric screening in two-dimensional insulators: Implications for excitonic and impurity states in graphane, Phys. Rev. B **84**, 085406 (2011).

[3] T. C. Berkelbach, M. S. Hybertsen, and D. R. Reichman, Theory of neutral and charged excitons in monolayer transition metal dichalcogenides, Physical Review B **88**, 045318 (2013).

[4] A. Chernikov, T. C. Berkelbach, H. M. Hill, A. Rigosi, Y. Li, O. B. Aslan, D. R. Reichman, M. S. Hybertsen, and T. F. Heinz, Exciton binding energy and nonhydrogenic Rydberg series in monolayer WS_2, Physical review letters **113**, 076802 (2014).

[5] M. Danovich, D. A. Ruiz-Tijerina, R. J. Hunt, M. Szyniszewski, N. D. Drummond, and V. I. Fal'ko, Localized interlayer complexes in heterobilayer transition metal dichalcogenides, Physical Review B **97**, 195452 (2018).

[6] I. C. Gerber, E. Courtade, S. Shree, C. Robert, T. Taniguchi, K. Watanabe, A. Balocchi, P. Renucci, D. Lagarde, X. Marie, and B. Urbaszek, Interlayer excitons in bilayer MoS_2 with strong oscillator strength up to room temperature, Physical Review B **99**, 1 (2019).

[7] A. Kormányos, G. Burkard, M. Gmitra, J. Fabian, V. Zólyomi, N. D. Drummond, and V. Falâ€™ko, k· p theory for two-dimensional transition metal dichalcogenide semiconductors, 2D Materials **2**, 022001 (2015).

[8] V. Savona, L. Andreani, P. Schwendimann, and A. Quattropani, Quantum well excitons in semiconductor microcavities: Unified treatment of weak and strong coupling regimes, Solid State Communications **93**, 733 (1995).

[9] N. U. Din, V. Turkowski, and T. S. Rahman, Ultrafast charge dynamics and photoluminescence in bilayer MoS_2, 2D Materials **8**, 025018 (2021).

[10] Z. Nie, R. Long, L. Sun, C.-C. Huang, J. Zhang, Q. Xiong, D. W. Hewak, Z. Shen, O. V. Prezhdo, and Z.-H. Loh, Ultrafast carrier thermalization and cooling dynamics in few-layer MoS_2, ACS nano **8**, 10931 (2014).

[11] M. Palummo, M. Bernardi, and J. C. Grossman, Exciton radiative lifetimes in two-dimensional transition metal dichalcogenides, Nano letters **15**, 2794 (2015).

[12] H. Wang, C. Zhang, and F. Rana, Ultrafast dynamics of defect-assisted electron–hole recombination in monolayer MoS_2, Nano letters **15**, 339 (2015).

[13] V. Shahnazaryan, I. Iorsh, I. A. Shelykh, and O. Kyriienko, Exciton-exciton interaction in transition-metal dichalcogenide monolayers, Phys. Rev. B **96**, 115409 (2017).

[14] E. A. Pogna, M. Marsili, D. De Fazio, S. Dal Conte, C. Manzoni, D. Sangalli, D. Yoon, A. Lombardo, A. C. Ferrari, A. Marini, *et al.*, Photo-induced bandgap renormalization governs the ultrafast response of single-layer mos2, ACS nano **10**, 1182 (2016).

[15] C. Trovatello, F. Katsch, Q. Li, X. Zhu, A. Knorr, G. Cerullo, and S. Dal Conte, Disentangling many-body effects in the coherent optical response of 2d semiconductors, Nano Letters **22**, 5322 (2022).

[16] L. Zhang, F. Wu, S. Hou, Z. Zhang, Y.-H. Chou, K. Watanabe, T. Taniguchi, S. R. Forrest, and H. Deng, Van der waals heterostructure polaritons with moiré-induced nonlinearity, Nature **591**, 61 (2021).

[17] I. Hughes and T. Hase, *Measurements and their uncertainties: a practical guide to modern error analysis* (OUP Oxford, 2010).

[18] H. Deng, H. Haug, and Y. Yamamoto, Exciton-polariton Bose-Einstein condensation, Reviews of Modern Physics **82**, 1489 (2010).

[19] R. Emmanuele, M. Sich, O. Kyriienko, V. Shahnazaryan, F. Withers, A. Catanzaro, P. Walker, F. Benimetskiy, M. Skolnick, A. Tartakovskii, *et al.*, Highly nonlinear trion-polaritons in a monolayer semiconductor, Nature communications **11**, 1 (2020).

[20] K. W. Song, S. Chiavazzo, and O. Kyriienko, Microscopic theory of nonlinear phase space filling in polaritonic lattices (2022).

[21] D. Erkensten, S. Brem, and E. Malic, Exciton-exciton interaction in transition metal dichalcogenide monolayers and van der waals heterostructures, Phys. Rev. B **103**, 045426 (2021).

[22] E. Ivchenko, M. Kaliteevski, A. Kavokin, and A. Nesvizhskii, Reflection and absorption spectra from microcavities with resonant bragg quantum wells, JOSA B **13**, 1061 (1996).

[23] O. Kyriienko, E. B. Magnusson, and I. A. Shelykh, Spin dynamics of cold exciton condensates, Phys. Rev. B **86**, 115324 (2012).

[24] C. Ciuti, V. Savona, C. Piermarocchi, A. Quattropani, and P. Schwendimann, Role of the exchange of carriers in elastic exciton-exciton scattering in quantum wells, Physical Review B **58**, 7926 (1998).

[25] L. Butov, Cold exciton gases in coupled quantum well structures, Journal of Physics: Condensed Matter **19**, 295202 (2007).

[26] C. Schindler and R. Zimmermann, Analysis of the exciton-exciton interaction in semiconductor quantum wells, Physical Review B **78**, 045313 (2008).

[27] R. Zimmermann and C. Schindler, Exciton–exciton interaction in coupled quantum wells, Solid state communications **144**, 395 (2007).

[28] M. Combescot, O. Betbeder-Matibet, and F. Dubin, The many-body physics of composite bosons, Physics Reports **463**, 215 (2008).

[29] J. Gu, V. Walther, L. Waldecker, D. Rhodes, A. Raja, J. C. Hone, T. F. Heinz, S. Kéna-Cohen, T. Pohl, and V. M. Menon, Enhanced nonlinear interaction of polaritons via excitonic Rydberg states in monolayer WSe_2, Nature Communications **12**, 10.1038/s41467-021-22537-x (2021), arXiv:1912.12544.

[30] A. Delteil, T. Fink, A. Schade, S. Höfling, C. Schneider, and A. İmamoğlu, Towards polariton blockade of confined exciton–polaritons, Nature materials **18**, 219 (2019).

[31] G. Muñoz-Matutano, A. Wood, M. Johnsson, X. Vidal, B. Q. Baragiola, A. Reinhard, A. Lemaître, J. Bloch, A. Amo, G. Nogues, *et al.*, Emergence of quantum correlations from interacting fibre-cavity polaritons, Nature materials **18**, 213 (2019).
